# Interplay of phosphate and carbonate ions with flavin photosensitizers in photodynamic inactivation of bacteria

**DOI:** 10.1371/journal.pone.0253212

**Published:** 2021-06-11

**Authors:** Daniel Bernhard Eckl, Stefanie Susanne Eben, Laura Schottenhaml, Anja Eichner, Rudolf Vasold, Andreas Späth, Wolfgang Bäumler, Harald Huber

**Affiliations:** 1 Department of Microbiology, University of Regensburg, Regensburg, Germany; 2 Clinic and Polyclinic of Dermatology, University Hospital Regensburg, Regensburg, Germany; 3 Department of Organic Chemistry, University of Regensburg, Regensburg, Germany; 4 TriOptoTec GmbH, Regensburg, Germany; Universidade de Aveiro, PORTUGAL

## Abstract

Photodynamic inactivation (PDI) of pathogenic bacteria is a promising technology in different applications. Thereby, a photosensitizer (PS) absorbs visible light and transfers the energy to oxygen yielding reactive oxygen species (ROS). The produced ROS are then capable of killing microorganisms via oxidative damage of cellular constituents. Among other PS, some flavins are capable of producing ROS and cationic flavins are already successfully applied in PDI. When PDI is used for example on tap water, PS like flavins will encounter various ions and other small organic molecules which might hamper the efficacy of PDI. Thus, the impact of carbonate and phosphate ions on PDI using two different cationic flavins (FLASH-02a, FLASH-06a) was investigated using *Staphylococcus aureus* and *Pseudomonas aeruginosa* as model organisms. Both were inactivated *in vitro* at a low light exposure of 0.72 J cm^-^^2^. Upon irradiation, FLASH-02a reacts to single substances in the presence of carbonate or phosphate, whereas the photochemical reaction for FLASH-06a was more unspecific. DPBF-assays indicated that carbonate and phosphate ions decreased the generation of singlet oxygen of both flavins. Both microorganisms could be easily inactivated by at least one PS with up to 6 log_10_ steps of cell counts in low ion concentrations. Using the constant radiation exposure of 0.72 J cm^-2^, the inactivation efficacy decreased somewhat at medium ion concentrations but reached almost zero for high ion concentrations. Depending on the application of PDI, the presence of carbonate and phosphate ions is unavoidable. Only upon light irradiation such ions may attack the PS molecule and reduce the efficacy of PDI. Our results indicate concentrations for carbonate and phosphate, in which PDI can still lead to efficient reduction of bacterial cells when using flavin based PS.

## Introduction

Flavins in general are based on a heterocyclic 7,8-dimethylisoalloxazine and appear as yellow substances with excellent water solubility [1,2]. The most important flavin taken up by the animal digestive tract is riboflavin, also known as vitamin B_2_ or as food additive E101 [3]. Unlike riboflavin, FMN (flavin mononucleotide) and FAD (flavin adenine dinucleotide) are commonly associated to proteins as cofactors. The first isolation of a flavin, called lactochrome, was executed in 1879 from cow’s milk by the English chemist A. W. Blyth [1,4].

FMN fulfills for example important functions in biochemical pathways [5] as a coenzyme for oxidoreductases [2] and plays a major role in aerobic cellular respiration. FMN functions as an electron carrier in the complex I of the respiratory chain [6]. In addition, flavins play an important role in the bioluminescence of *Aliivibrio fischeri* [7]. In 1949 Arthur W. Galston discovered that riboflavins are capable of oxidizing indolacetic acid in plants by the requirement of oxygen and light, which also led to growth deficits in plants [8].

Flavins are also well known for forming several redox states. They either occur in oxidized form, as semiquinone or hydroquinone. Each redox state of the flavins has a distinct absorbance spectrum that can be used to differentiate between each state [1]. Flavin semiquinones as well as hydroquinones are likely to react with molecular oxygen resulting in the oxidized form that is known to be stable in standard atmospheric condition [1].

The oxidation of flavin molecules via generation of a light-induced triplet state was shown elsewhere [9] as well as singlet oxygen generation of riboflavin [10]. Eichler *et al*. observed that endogenous flavins are capable of producing ROS intracellularly [11]. In this case, the authors could not detect harmful effects and discussed a protective effect of flavin binding proteins. Although such endogenous flavins like riboflavin are capable of efficiently producing singlet oxygen [12–15], a sufficient inactivation of bacteria was not observed [16,17].

The measurement of the quantum efficiency of singlet oxygen generation (Φ_Δ_) of riboflavin, FMN and FAD by Baier *et al*. in 2006 [14] paved the way for the application of riboflavin as a new photosensitizer (PS) in photodynamic inactivation of microorganisms (PDI).

Thus, the same research group decided to change the disadvantage of riboflavin, namely the lack of a positive net charge hampering the attachment of riboflavin to bacterial cells. The synthesis of cationic riboflavin molecules enabled a highly effective PDI for different bacterial strains [18,19]. A close cell attachment or cellular uptake of a PS is mandatory because singlet oxygen shows a short diffusion range of less than 100 nm in cellular environments [20,21].

Flavins, either cationic or not, are molecules with interesting properties as PS. These molecules are biodegradable and therefore considered safe for human use [18,22]. Limited water solubility hinders gastrointestinal uptake [23] and high doses do not lead to side effects [24]. These properties allow flavins to be used in a wide range of applications ranging from humans to animal products as well as applications in the environment. However, potential fields of application for flavin PS like the human skin, the oral cavity, water disinfection and inanimate surfaces show the presence of different cations such as sodium ions as well as potassium ions and anions like chloride, carbonate and phosphate.

Thus, the present study aimed to investigate PDI with cationic flavin PS, exemplarily regarding the potential impact of sodium carbonate and sodium phosphate in four different concentrations. To unveil the potential effects of such ions, the experiments were performed at low light exposure of 0.72 J cm^-^^2^. Biological logarithmic reduction data were supplemented with assays of PS attachment towards cells, chemical analysis and physical measurements.

## Material and methods

### Bacterial strains

Bacterial strains were purchased from the German Collection of Microorganisms and cell culture lines (DSMZ, Braunschweig, Germany). As a Gram-positive representative *Staphylococcus aureus* DMZ 1104 was used, as Gram-negative model organism *Pseudomonas aeruginosa* DSMZ 1117. All organisms were grown on Mueller-Hinton-Agar [25] at 37°C.

### Preparation of the ionic solutions

Solutions were prepared as stock solutions with a concentration of 150, 15, 1.5 and 0.15 mmol l^-1^. As a solvent ultra-pure water with a conductance > 18 Ω was used, hereafter called H_2_O. After preparation, solutions were stored in plugged, sealed, gas tight serum bottles under nitrogen atmosphere in the dark at room temperature. pH value was adjusted to 7 with phosphoric acid for sodium phosphate solutions while for sodium carbonate HCl was used for adjustment. Sodium phosphate (Na_3_PO_4_) was obtained from Merck KGaA, Darmstadt, Germany while sodium carbonate (Na_2_CO_3_) was bought from Sigma-Aldrich, St. Louis, MO, USA, both in analytical grade.

### Photosensitizer

**Fig 1:**
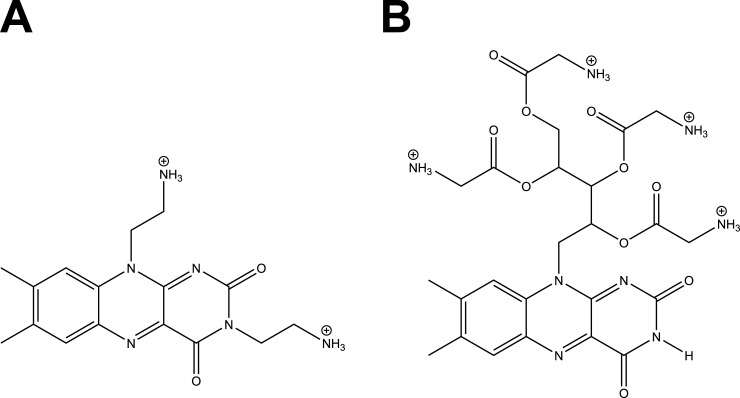
Structure of the used PS. (A) shows the structure of FLASH-02a, (B) shows the structure of FLASH-06a. The counterions of the substances were in both cases chloride anions.

The PS were flavin derivatives designated as FLASH-02a and FLASH-06a [26]. Both PS were purchased from the TriOptoTec GmbH (Regensburg, Germany) with a minimum dye content of 97%. The structure of the PS is displayed in Fig 1A for FLASH-02a and Fig 1B for FLASH-06a, FLASH-02a is a doubly charged cation with a rather small side chain compared with the fourfold charged cationic PS FLASH-06a with remarkably larger side chains. The positive charge of both PS allows the molecules to be located in the vicinity of bacteria enabling efficient inactivation.

### Spectroscopy

In order to investigate the chemical interactions of ions and PS at various concentrations, absorption spectroscopy in the visible spectral range (VIS) was performed. Assays were composed out of a total volume of 200 μl with PS concentrations ranging from 0 to 50 μmol l^-1^ and ionic solutions in concentrations of 0.075 mmol l^-1^ to 75 mmol l^-1^. Spectra measurement took place in 96-well plates utilizing a BMG Labtec plate reader (BMG Labtec, Ortenberg, Germany). Spectra were measured prior and after irradiation with up to 10.8 J cm^-2^, equivalent to 10 min of irradiation. The obtained percentage transmission was plotted with OriginLab 2019b (Northampton, USA). In order to analyse isosbestic points, the apparent coefficient of variation was calculated as follows

$$CV=\frac{\sigma (\lambda )}{A(\lambda )}\times 100$$

*CV*: Apparent coefficient of variation

*σ*(*λ*): Standard deviation at wavelength *λ*

*A*(*λ*): Absorption at wavelength *λ*

Whenever an intersection of the spectra occurred and the apparent coefficient was below 2.5% [27], an isosbestic point was assumed.

### DPBF-assays

DPBF-assays aimed for a qualitative statement whether the photophysical properties of the dye are hindered in the presence of ions. 1,3-Diphenylisobenzofurane (DPBF) was purchased from Sigma-Aldrich with a minimum dye content of 97%. Each assay was composed as follows: 200 μl in dark 96-well plates (Sarstedt AG & Co. KG, Nuembrecht, Germany) contained a concentration of 0 to 50 μmol l^-1^ PS, 75, 7.5, 0.75 or 0.075 mmol l^-1^ of the corresponding ion and 500 μmol l^-1^ DPBF (dissolved in ≥99% methanol, analytical grade). The DPBF solution was prepared immediately before each experiment. Controls consisted out of 100 μl ionic solution in appropriate concentration and 100 μl methanol. Internal references contained 100 μl H_2_O and 100 μl methanol with 500 μmol l^-1^ DPBF. Each value for each condition was obtained as the mean value of three independent replicates. The assays were measured after 0, 1, 2, 3, 4, 5 and 10 s of irradiation with the beforehand mentioned light source utilizing a fluorescence plate reader, purchased from BMG Labtech. Excitation wavelength was set to 411 nm while emission was detected at 451 nm. Values obtained for the internal reference were set to 1, relative fluorescence was calculated as ratios to the control and displayed in per cent via OriginLab 2019b. A statistical analysis of the retrieved values was performed afterwards as explained in S1 File.

### Light source

Irradiation of all samples was performed with a blue light emitting prototype containing a neon tube with an emission range from about 380–470 nm (BlueV, Medizintechnik Herbert Waldmann GmbH & Co. KG, Villingen-Schwenningen, Germany). Intensity was measured with a thermal sensor (Nova 30 A-P-SH, Ophir-Spiricon, North Logan, UT, USA). All irradiation steps were carried out at a light irradiance of 18 mW cm^-2^.

### Photodynamic treatment of bacteria

Bacteria were taken from the agar plates and suspended in H_2_O. Optical density was adjusted to 0.6 at 600 nm utilizing a photometer (Ultrospec 10, Amersham Biosciences, Little Chalfont, UK). After density adjustment, 1 ml of the bacterial suspension was transferred to 1.5 ml reaction tubes and centrifuged at 13,000 x g for 7 min. Subsequently, supernatant was discarded and the pellet was resuspended in H_2_O. Centrifugation was repeated and mixed thoroughly with 1000 μl of ionic solution prepared as mentioned above. 25 μl of the prepared suspension were then mixed with 25 μl of PS-solution in ascending concentrations, incubated for 10 min in absolute darkness and irradiated with a constant energy of 0.72 J cm^-2^ (corresponding to an illumination time of 40 s) afterwards.

20 μl of this reacting solution were transferred to 180 μl preheated Mueller-Hinton bouillon and cultivated at 37°C for 48 h. A plate reader measured the optical density at 600 nm which was used to calculate the bacterial reduction as described in detail elsewhere [28]. In principle, the applied method concerning cultivation and evaluation of the microbial reduction is a adaption of a method initially described by Bechert and co-workers [29] and has been successfully used for example by Bruenke *et al*.[30]. Doubling time was calculated for an optical density of 0.2 and 0.4. All light sensitive parts of the procedure were conducted at low light conditions as investigated elsewhere [31]. Obtained inactivation values, which were obtained from three independent measurements, were analyzed statistically as mentioned in S2–S4 Files.

### Binding assays

To investigate binding of flavins to bacteria, optical density at 600 nm was adjusted to 0.6. 500 μl cell suspensions were placed in 1.5 ml reaction tubes, centrifuged as mentioned before and washed in H_2_O. The pellet was suspended in 500 μl of ionic solution and 500 μl of PS with a concentration of 100 μmol l^-1^ and incubated for 10 min in the dark. After incubation, the cells were centrifuged at 4,500 x g for 10 min. The supernatant was transferred into a cuvette and measured against a control. Measurements were carried out at 444 nm for FLASH-02a and 446 nm for FLASH-06a, respectively. Each condition was tested independently thrice leading to three independent values. A statistical analysis of the binding assays was performed and is shown in S5 File.

### Statistical analysis

Data from DPBF-assays were collected in three independent replicates, while values of the binding assays and logarithmic reduction rates from the photodynamic inactivation experiments were obtained from three biological replicates of each tested condition. In order to investigate the data statistically the values of the replicates were compared between the tested conditions and p-values were calculated via unpaired, two-tailed t-tests assuming normal distribution. Events were considered statistically significant for p < 0.05. When p was < 0.01, events were considered highly significant, p < 0.001 indicated extremely significant events. The calculated p-values are given in S1-S5 Supporting information, the values of the replicates are given in the S3–S7 Datasets.

## Results

### Photochemical alterations of the PS

**Fig 2:**
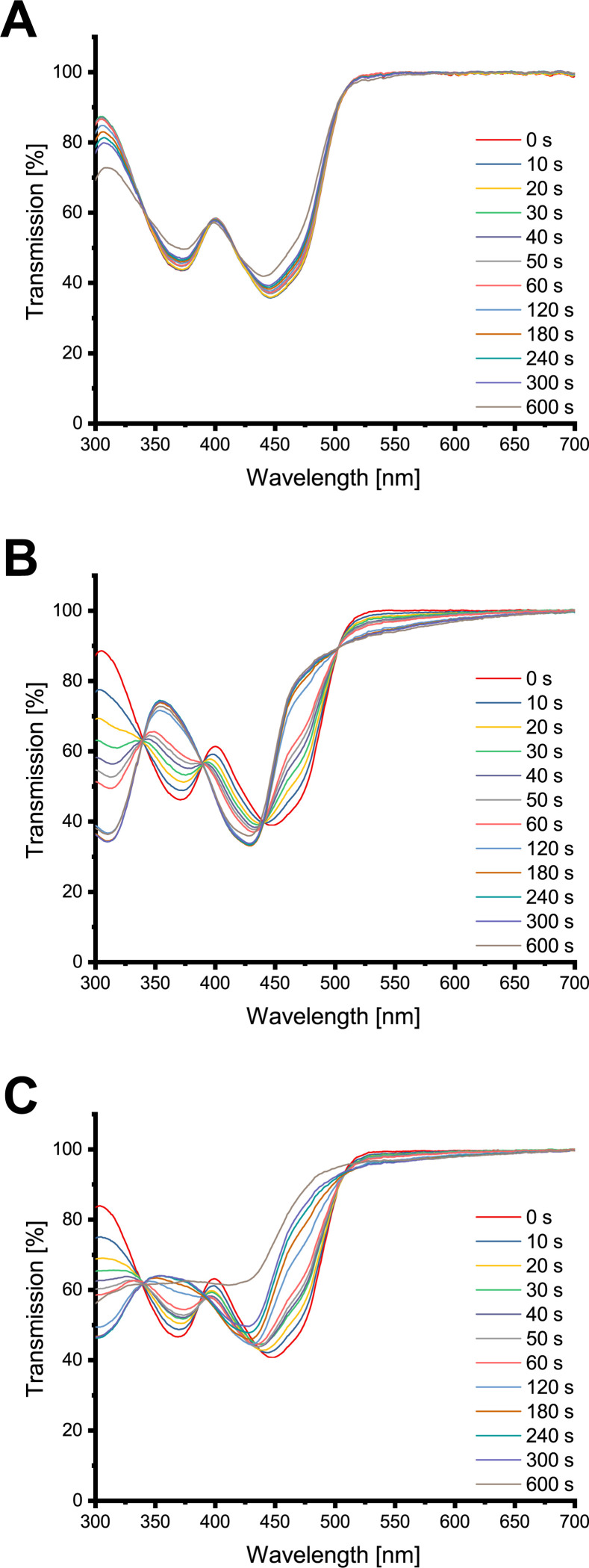
Transmission spectra of FLASH-02a. Y axes indicate the transmission in per cent, x axes the wavelength in nm. Various colors of the spectra indicate different irradiation times. (A) shows the results for H_2_O, (B) for 75 mmol l^-1^ sodium phosphate and (C) for 75 mmol l^-1^ sodium carbonate.

FLASH-02a showed photodegradation upon light exposure as indicated by measuring the transmission of the PS in H_2_O after the application of 10.8 J cm^-2^ (equal to 10 min of irradiation), equivalent to a loss of concentration of about 17% (Fig 2A). The presence of phosphate and carbonate ions in solutions caused an alteration of the spectra depending on ion concentration and irradiation time. The photochemically changed flavins show isosbestic points at 339 nm (CV = 1.55%), 389 (CV = 0.96%), 440 (CV = 2.25%) and 503 nm (CV = 1.24%) for phosphate (Fig 2B) and 339 nm for carbonate with CV equal to 1.01% (Fig 2C). The acquired transmission data are listed in detail in the S1 Dataset.

**Fig 3:**
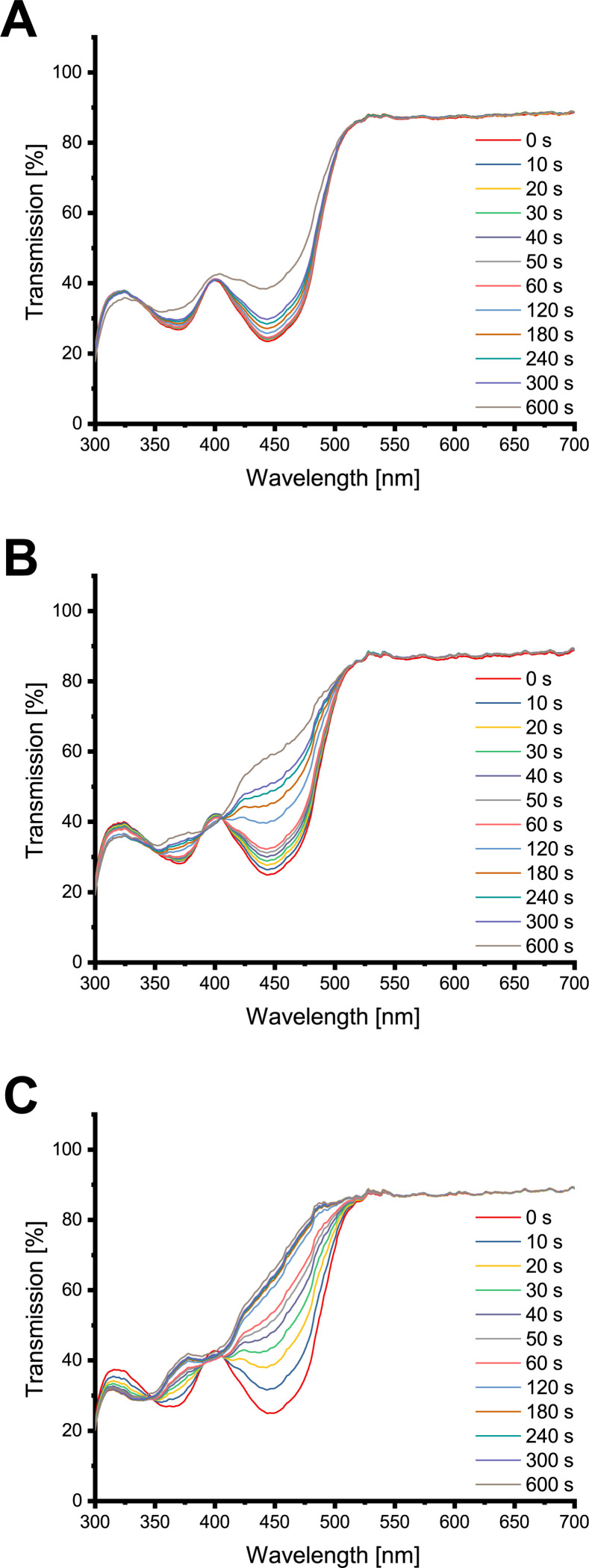
Transmission spectra of FLASH-06a. Y axes indicate the transmission in per cent, x axes the wavelength in nm. Various colors of the spectra indicate different irradiation times. (A) shows the results for H_2_O, (B) for 75 mmol l^-1^ sodium phosphate and (C) for 75 mmol l^-1^ sodium carbonate.

FLASH-06a was far more affected by photobleaching most likely due to its large side chains. After the application of 10.8 J cm^-2^ (equal to 10 min of irradiation), around 34% of the PS were degraded in the water control (Fig 3A). FLASH-06a in combination with phosphate showed isosbestic points at 348 (CV = 1.24%), 389 (CV = 0.68%) and 405 nm (CV = 0.83%) (Fig 3B). Interestingly, the isosbestic points for carbonate were about the same wavelength as for phosphate with 346 nm (CV = 1.17%), 393 nm (CV = 1.75%) and 402 nm (CV = 1.95%) (Fig 3C). However, chemical reaction was more pronounced with less light exposure for carbonate. The transmission data are shown in the S2 Dataset.

### Singlet oxygen production with and without ions

**Fig 4:**
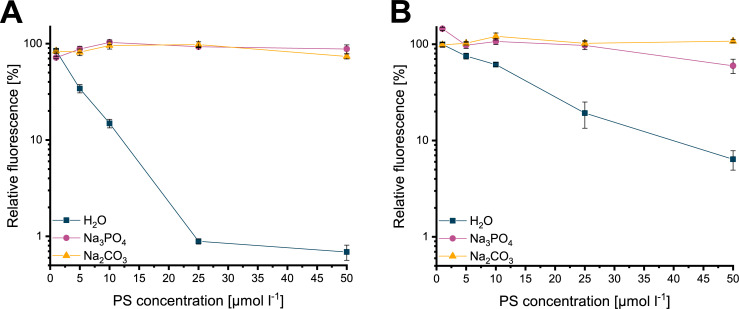
Results for the DPBF assays. Relative fluorescence in presence of FLASH-02a is shown in (A) and FLASH-06a in (B) after 10 s of irradiation. Different colors of the lines and symbols indicate the used environment. In this experiment, 75 mmol l^-1^ of sodium phosphate or sodium carbonate were applied. Y axes indicate the relative fluorescence in percent referenced to the DPBF control, X axes indicate the PS concentration in μmol l^-1^. Error bars display the calculated standard deviation of the triplicates.

The singlet oxygen production was monitored by relative fluorescence of DPBF. Without the addition of ions, relative DPBF fluorescence decreased after 10 s of irradiation with 18 mW cm^-2^ (equal to 0.18 J cm^-2^) and reached values below 1% for the maximum concentration of 50 μmol l^-1^ FLASH-02a (Fig 4A). The results for the H_2_O control of the DPBF assays carried out with FLASH-02a diverge all significantly from each other–except for a comparison of the highest two PS concentrations (detailed results are displayed in S1 File).

However, with increasing concentrations of sodium phosphate or sodium carbonate, less singlet oxygen was generated as indicated by the constant relative DPBF fluorescence. The statistical analysis also showed in nearly all compared conditions non-significant events, whereas the detailed results are given in S1 File. When using FLASH-06a without the addition of ions, relative DPBF fluorescence decreased after 10 s of irradiation with 18 mW cm^-2^ (0.18 J cm^-2^) and reached minimum values below 7% for the maximal concentration of 50 μmol l^-1^ FLASH-06a (Fig 4B). Again, a comparison of all tested conditions showed that all values were significantly distinct from each other, with the comparison of the highest two PS concentrations being the only exception. With increasing concentrations of sodium phosphate, less singlet oxygen was generated. However, the obtained values for 1 and 5 μmol l^-1^ of FLASH-06a differ from the other PS concentrations significantly (S1 File). The same result was observed for increasing concentrations of sodium carbonate, in which none of the tested conditions does vary significantly from each other (S1 File). Values above 100% indicate that the photobleaching effect due to residual light of the control exceeded the decrease in fluorescence caused by singlet oxygen production of the photosensitizer. Measured values, means and standard deviation are shown in the S3 Dataset.

### Photodynamic inactivation of bacteria with and without ions

**Fig 5:**
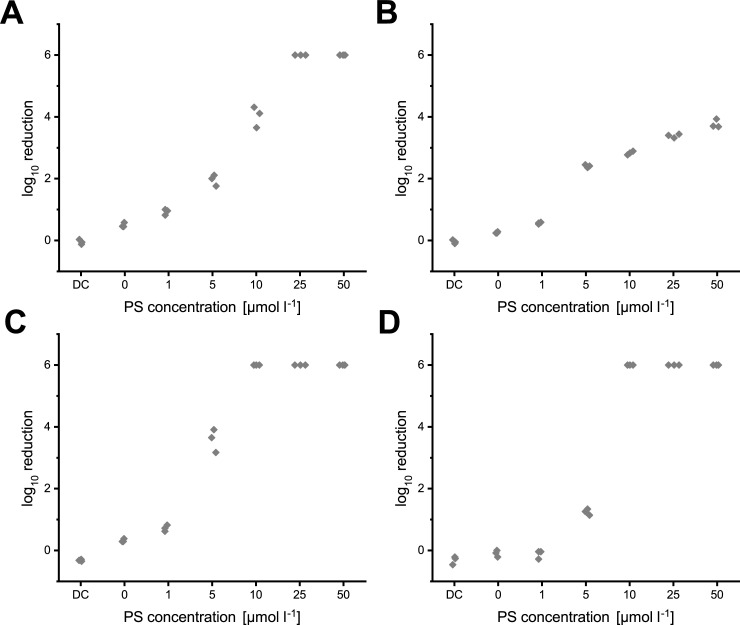
Results for the photodynamic inactivation without the application of ions. Logarithmic reduction values of *S*. *aureus* with FLASH-02a (A) and FLASH-06a (B) compared to the logarithmic reduction of *P*. *aeruginosa* treated with FLASH-02a (C) and FLASH-06a (D). Y axis of the graphs indicate the decadic logarithmic reduction while the x axis displays the different concentrations of the applied PS with DC indicating the dark control (no irradiation, 50 μmol l^-1^ PS). As each experiment was carried out by n = 3, each dot represents one single value from a single experiment.

PDI experiments in H_2_O revealed a higher inactivation efficiency for *Pseudomonas aeruginosa* (Fig 5C and 5D) compared to *Staphylococcus aureus* for both PS (Fig 5A and 5B). In general, FLASH-06a was less efficient in the inactivation of bacterial cells, for *S*. *aureus* the inactivation did not exceed four orders of magnitude (Fig 5B). However, besides the treatment of *S*. *aureus* with FLASH-06a, both bacteria could be inactivated to the lower limit of detection of six orders of magnitudes. Each obtained logarithmic reduction value for each experiment is displayed in the S4 Dataset. All tested conditions diverge significantly from each other except for concentrations of FLASH-06a below 5 μmol l^-1^ for *P*. *aeruginosa*. Detailed statistics are given in S2 File.

When comparing the photodynamic inactivation for *S*. *aureus* in H_2_O, sodium carbonate and sodium phosphate, dark control (DC), light control (0 μmol l^-1^) and 1 μmol l^-1^ of any PS, no noteworthy reduction of the number of viable bacterial cells was obtained. Even with increasing PS concentrations up to 50 μmol l^-1^, no efficient inactivation was achieved for 75 and 7.5 mmol l^-1^ of the ions at the constant light exposure of 0.72 J cm^-2^ (40 s of irradiation).

**Fig 6:**
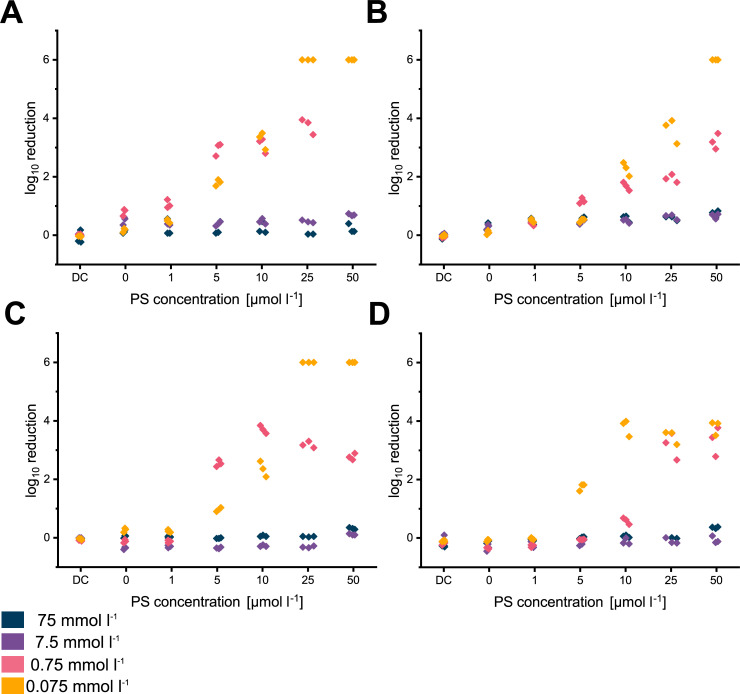
Logarithmic inactivation for *S*. *aureus* data obtained for each experiment displayed as scatter plot. (A) shows results for sodium phosphate and FLASH-02a, (B) displays the results for sodium carbonate and FLASH-02a. Obtained values for FLASH-06a are displayed in (C) for sodium phosphate and (D) for sodium carbonate. Y axes indicate the decadic logarithmic reduction and x axes indicate the PS concentration in μmol l^-1^ or the dark control (DC). Different colors of the dots indicate the various sodium carbonate or sodium phosphate concentrations.

Only if concentrations of sodium phosphate were reduced to at least 0.75 mmol l^-1^ and 5 μmol l^-1^ of FLASH-02a (Fig 6A) or FLASH-06a (Fig 6C), logarithmic reduction started to exceed one order of magnitude. For sodium carbonate and FLASH-02a, this critical point was reached for 10 μmol l^-1^ of respective PS with 0.75 mmol l^-1^ of sodium carbonate. Therefore, PDI had a better efficiency with phosphate, while carbonate seemed to have a much more detrimental effect on the system (Fig 6A and 6C vs. Fig 6B and 6D). For *S*. *aureus*, the best inactivation results were obtained when ion concentrations were as low as possible with PS concentrations from 25 to 50 μmol l^-1^. A statistical analysis of the logarithmic reduction values of the various applied conditions is given in S3 File. Measured logarithmic reduction values for each experiment are displayed in the S5 Dataset.

**Fig 7:**
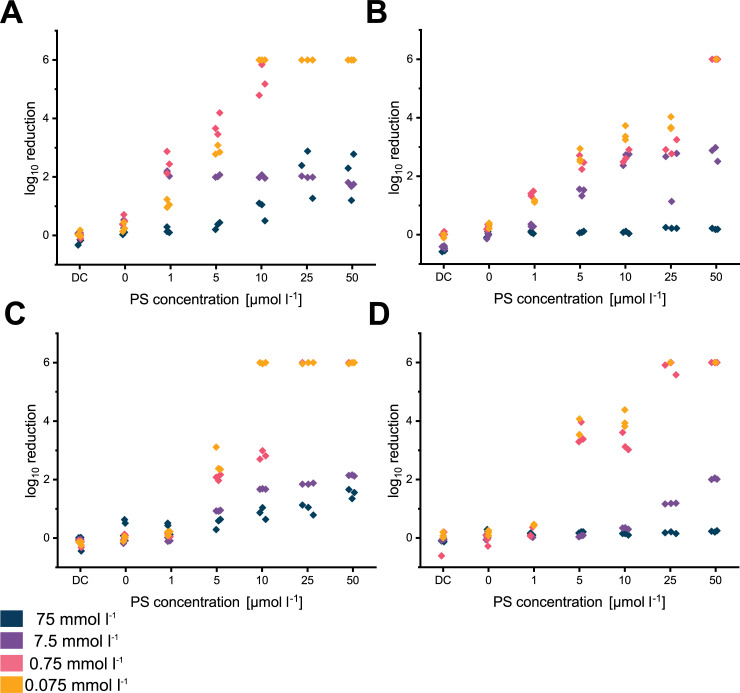
Logarithmic inactivation for *P*. *aeruginosa* data obtained for each experiment displayed as scatter plot. (A) shows results for sodium phosphate and FLASH-02a, (B) displays the results for sodium carbonate and FLASH-02a. Obtained values for FLASH-06a are displayed in (C) for sodium phosphate and (D) for sodium carbonate. Y axes indicate the decadic logarithmic reduction and x axes indicate the PS concentration in μmol l^-1^ or the dark control (DC). Different colors of the dots indicate the various sodium carbonate or sodium phosphate concentrations.

The observations were quite similar for the Gram-negative organism *P*. *aeruginosa*. H_2_O, sodium carbonate and sodium phosphate did not lead to a reduction of the dark control (DC), light control (0 μmol l^-1^) and 1 μmol l^-1^ exceeding one order of magnitude. Again, increasing PS concentrations did not result in an efficient inactivation exceeding three orders of magnitude for 75 and 7.5 mmol l^-1^ of both tested solutions. In contrast to the results obtained for *S*. *aureus*, the Gram-negative strain was eradicated with more than 6 orders of magnitude for 0.075 mmol l^-1^ of sodium phosphate (Fig 7A and 7C) with PS concentrations as low as 10 μmol l^-1^. The results of the statistical analysis of the obtained logarithmic reduction values for *P*. *aeruginosa* are included in S4 File. Inactivation of *P*. *aeruginosa* in the presence of sodium carbonate was less efficient than for sodium phosphate which was in good agreement with results obtained for *S*. *aureus* (Fig 7B and 7D). In general, inactivation with FLASH-02a was more efficient (Figs 6A, 6B, 7A and 7B) than with FLASH-06a (Figs 6C, 6D, 7C and 7D). Measured logarithmic reduction values for each experiment are deposited in the S6 Dataset.

Additionally, the presence of carbonates and phosphates did not alter the binding behavior of the PS in a detrimental manner, the amount of PS attached to bacterial cells did either not diverge significantly from the H_2_O control or was in some cases significantly higher when ionic solutions were applied. The results of a statistical analysis are included in S5 File. The mean calculated amounts and the corresponding standard deviation of bound PS to the respective cells are shown in Table 1. The results of each measurement are included in the S7 Dataset.

**Table 1:** Results of the binding assays.

PS	Ions	c (salt) [mmol l^-1^]	c(PS) bound to *S*. *aureus* [μmol l^-1^]	Standard deviation	c(PS) bound to *P*. *aeruginosa* [μmol l^-1^]	Standard deviation
FLASH-02a	H_2_O		31.29	0.91	30.90	0.29
	Na_2_CO_3_	75	31.77	1.72	28.51	1.09
	Na_2_CO_3_	0.075	33.11	1.41	35.40	0.66
	Na_3_PO_4_	75	31.39	0.35	39.68	0.50
	Na_3_PO_4_	0.075	32.67	1.38	35.60	0.42
FLASH-06a	H_2_O		25.76	1.02	27.71	1.13
	Na_2_CO_3_	75	27.53	1.41	28.29	0.49
	Na_2_CO_3_	0.075	29.11	0.98	29.96	0.75
	Na_3_PO_4_	75	28.12	1.01	27.64	1.93
	Na_3_PO_4_	0.075	29.76	0.97	31.28	0.32

## Discussion

The results of the present investigation show some obstacles as ions like carbonate and phosphate can hamper PDI of flavin PS. Ions in combination with the herein used PS change the mechanism in chemical, physical and biological manners.

The spectra without ions showed a photobleaching effect that was more pronounced for FLASH-06a compared to FLASH-02a, most likely due to the chemical nature of the molecules due to larger side chains of FLASH-06a. Degradation effects without involvement of ions have been described for other flavins previously [32,33].

However, the presented study showed that phosphate and carbonate react by a light-based mechanism with the used flavin PS altering the chemical structure. Time dependent spectra showed isosbestic points under addition of carbonate and phosphate. Isosbestic points in general are strong hints at mass conservation, indicating a variety of compounds, most likely even the involvement of just two substances [34].

Even though all ion treated PS with isosbestic points meet the requirement according to the definition of Thomas and Burgess [27] the isosbestic points are not sharp and clearly defined in a strict sense. Shifts in isosbestic points are either caused by different molar extinction coefficients due to the experimental condition or the contribution of a third, unknown substance [35], experimentally shown by Harris and Bashford [36].

It is known that riboflavin derivatives undergo chemical changes upon light irradiation, resulting in several isosbestic points in the absorption spectra [37–39]. We tentatively assume that degradation of FLASH-02a in the presence of phosphate occurs via cleavage of the side chain to lumichrome [32] possibly followed by an unspecific degradation as observed for example in dairy products [40]. However, for carbonate the transmission spectra are quite distinct from the ones of phosphate, indicating the involvement of more than one substance. Therefore, it is speculated that in this specific case after the side chain cleavage additionally to lumichrome lumiflavin is produced. Neither of the both assumed molecules are capable of producing singlet oxygen. Organisms encounter such degradation products on a daily basis without being harmed, as shown for example for human keratinocytes where no [18] or very low [22] toxicity was found. Although the structural discussion of FLASH-02a is more of a speculative nature, FLASH-06a presents with a clearer situation. It is hypothesized that the ester bonds of the side chains are at least partially hydrolyzed. The remaining scaffold of the former PS might then undergo a cyclization comparable to cyclodehydroriboflavin CDRF–similar to the photoaddition of riboflavin [32,41]. It has been described elsewhere that both carbonate and phosphate facilitate such processes [41]. However, carbonate promotes the cyclisation more than phosphate, which is also supported by the data shown in this publication. The work of Vaid *et al*. shows that the cyclization of riboflavin is depending on the ionic concentration of are in good consistency with the results presented here. The proposed CDRF-like end product is just as lumichrome or lumiflavin no longer capable of producing singlet oxygen as it was observed in this study by DPBF assays. Again, we observed a less detrimental effect of phosphate for singlet oxygen production compared to carbonate which is also supported by work from other researchers [41].

Additionally, this publication may impact PDI in general as well. Ubiquitous ions as well as other small molecules are frequently present when PDI is applied outside a laboratory setting. These substances may initiate photochemical reactions of the PS leading to the usage of the absorbed light energy for chemical reactions rather than for singlet oxygen production. Such effects decrease the efficacy of killing bacteria as shown here for flavin PS by reducing the amount of generated singlet oxygen. However, e.g. Kainz *et al*. demonstrated the formation of zinc(II)-cyclen-flavin complexes in combination with Fe/C nanoparticles [42]. The use of such methods could protect flavins from harmful effects caused by ions.

It is crucial to understand how ions influence PDI since a major aim is the application of photodynamic inactivation in environments outside the laboratory. Future fields of application include the treatment of wastewater [43,44], the disinfection of drinking water [45,46], decontamination of food [47], the reduction of the bacterial load on surfaces [48] or the decolonization of skin [49]. However, ions are not only included in environmental photodynamic inactivation, but sometimes even *in vitro* studies often use phosphate buffered saline or culture medium for PDI experiments that contain various ions or inhibitory substances [50–52]. Tap water or wastewater may contain various ions like Na^+^, Ca^2+^, Mg^2+^, and HCO_3_^−^ with a concentration to a few mmol l^-1^, whereas these ions can also be found on animal or human skin, in particular HCO_3_^−^ with concentrations of up to 4 mmol l^-1^ [53,54].

A literature screening concerning PS concentration and applied fluence reveals divergent parameters that were chosen for efficient inactivation. On the one hand, PDI was efficient of up to four orders of magnitude at low light doses and low porphyrin PS concentrations under ambient light conditions [31]. Yang *et al*. successfully inactivated *Propionibacterium acnes* with a curcumin PS by applying 0.09 J cm^-2^ and PS concentrations as low as 1.5 μmol l^-1^ [55]. Efficient inactivation was also shown with flavin PS at a concentration of 10 μmol l^-1^ and a light exposure of 1.5 J cm^-2^ [18]. Jemli and co-workers showed an efficient reduction of five orders of magnitude by using 10 μmol l^-1^ of methylene blue, rose Bengal or TMPyP with a light dose of 0.13 J cm^-2^ in an wastewater environment [43].

On the other hand, some studies used elevated PS concentrations and/or high light exposures [56–58]. Bactericidal action in wastewater for example was achieved with different PS in the μmol l^-1^ range at high light exposures up to a few hundred J cm^-2^ [43,45,59]. Carvalho *et al*. studied the application of PDI in the context of wastewater treatment, achieving a logarithmic reduction of three orders of magnitude. The study used a porphyrin PS at a concentration as low as 5 μmol l^-1^ and a light dose of 145.8 J cm^-2^ [44].

A review article [60] listed 19 animal studies in which bacteria were treated in wounded skin using different PS. It is striking that the light exposure shows high values ranging from 6–423 J cm^-2^ with a mean value of 163 J cm^-2^. Another study showed the difference of *in vitro* and *in vivo* application of PDI. When using a cationic Zn(II) phthalocyanine PS, MRSA was efficiently inactivated *in vitro* using PS concentrations of less than 1 μmol l^-1^ and a 48 J cm^-2^ light exposure [61]. For inactivation of MRSA in an animal wound model *in vivo* the authors had to increase PS concentration to 7.8 μmol l^-1^ and light exposure to about 300 J cm^-2^. Another research group showed the inactivation of clinically relevant organisms with polycationic PS in nutrient broth. Possibly due to inhibitory substances in the culture medium, light doses of up to 40 J cm^-2^ had to be applied [56].

In a publication concerning a phenalenone PS, organisms with importance in dentistry like *Enterococcus faecalis*, *Actinomyces naeslundii* or *Fusobacterium nucleatum* were efficiently inactivated. These experiments were carried out in PBS buffer with PS concentrations of 100 μmol l^-1^ and light doses of 72 J cm^-2^ [57]. Another study used quite similar organisms and PS in concentrations of up to 250 μmol l^-1^ applying light doses of up to 150 J cm^-2^ [58].

Therefore, the major problem behind these studies is that all used different PS, bacteria, PS concentrations, light sources, light irradiance and light doses. Although some adaptions are necessary when using different photosensitizers or bacteria, it is obvious that there are further elements impacting PDI. Higher light exposure and PS concentrations might be applied to counteract detrimental effects of various substances that are inevitably present in environmental settings. Four studies also assumed a connection between the presence of inhibitory substances and reduced inactivation efficacy [62–65]. Besides only inhibitory effects there are also enhancing substances known such as EDTA [65], sodium azide [66] and potassium iodine [67,68].

## Conclusion

The success of PDI as a new treatment against pathogens requires a successful application beyond standardized laboratory experiments. The impact of ubiquitous ions on the photodynamic mechanism may complicate the application under environmental conditions. As shown in this study, carbonate and phosphate alter the chemical structure of the PS leading to less singlet oxygen generation and hence reduced inactivation of bacteria. The lower the amount of additional material in PDI and the higher the applied light dose is, the better the inactivation. This study provides practical advice for future studies with flavin PS in the presence of phosphate and carbonate. Furthermore, this study stresses the inevitable importance of chemical analysis, physical measurements and investigations concerning bacterial inactivation prior to the application outside the laboratory.

## Supporting information

S1 Dataset Transmission values for FLASH-02a. (XLSX)

S2 Dataset Transmission values for FLASH-06a. (XLSX)

S3 Dataset Relative fluorescence values of each conducted DPBF assay with calculated standard deviation. (XLSX)

S4 Dataset Logarithmic reduction values of each experiment for the tested organisms in H_2_O. (XLSX)

S5 Dataset Logarithmic reduction values of each experiment for *S*. *aureus* in ionic solutions. (XLSX)

S6 Dataset Logarithmic reduction values of each experiment for *P*. *aeruginosa* in ionic solutions. (XLSX)

S7 Dataset Concentration measurements of attached PS to bacterial cells. (XLSX)

S1 File Statistical analysis of DPBF assays. (PDF)

S2 File Statistical analysis of inactivation data without ions. (PDF)

S3 File Statistical analysis of *Staphylococcus aureus* inactivation data. (PDF)

S4 File Statistical analysis of *Pseudomonas aeruginosa* inactivation data. (PDF)

S5 File Statistical analysis of binding assay data. (PDF)
