# Supplementary material for: Interplay of phosphate and carbonate ions with flavin photosensitizers in photodynamic inactivation of bacteria
Source: PLoS One. 2021 Jun 11;16(6):e0253212. doi: 10.1371/journal.pone.0253212 (PMC8195418; doi:10.1371/journal.pone.0253212)
Supplement: S1 File — (PDF) [file pone.0253212.s008.pdf]

Statistical analysis of DPBF assays

Method

In order to investigate the gathered data statistically, derived from each triplicate means and standard deviation were calculated. Significance between samples was calculated via unpaired, two-tailed t-tests assuming normal distribution. Events were considered statistically significant for $p < 0.05$ and marked in the following table with one asterisk. When p is < 0.01, events were considered highly significant and marked with two asterisks. Extremely significant events with $p < 0.001$ were marked with three asterisks. Non-significant events were marked with “Ns”, whenever a calculation of the corresponding p-value was not possible due to the limit of detection (6 orders of magnitude), the values were marked with “Nd”.

Results

Table 1: Results of the performed t-tests of the obtained values from DPBF assays. 06 indicates the photosensitizer FLASH-06a, 02 FLASH-02a. The salt (in a concentration of 75 mmol l-1) follows chemical nomenclature. The photosensitizer concentration is found in the last column of each condition, the values are given in µmol l-1. Significance is displayed as mentioned in the method section

**Condítition 1**			**Vs.**	**Condition 2**			**p**	**significance**
**06**	H2O	1		06	H2O	5	0,01038	*
**06**	H2O	1		06	H2O	10	0,00208	**
**06**	H2O	1		06	H2O	25	0,00015	***
**06**	H2O	1		06	H2O	50	0,00086	***
**06**	H2O	5		06	H2O	10	0,04985	*
**06**	H2O	5		06	H2O	25	0,00057	***
**06**	H2O	5		06	H2O	50	0,00155	**
**06**	H2O	10		06	H2O	25	0,00194	**
**06**	H2O	10		06	H2O	50	0,00064	***
**06**	H2O	25		06	H2O	50	0,08145	Ns
**06**	Na_3PO_4	1		06	Na_3PO_4	5	0,00229	**
**06**	Na_3PO_4	1		06	Na_3PO_4	10	0,00607	**
**06**	Na_3PO_4	1		06	Na_3PO_4	25	0,00674	**
**06**	Na_3PO_4	1		06	Na_3PO_4	50	0,00185	**
**06**	Na_3PO_4	5		06	Na_3PO_4	10	0,27317	Ns
**06**	Na_3PO_4	5		06	Na_3PO_4	25	0,95938	Ns
**06**	Na_3PO_4	5		06	Na_3PO_4	50	0,01564	*
**06**	Na_3PO_4	10		06	Na_3PO_4	25	0,31429	Ns
**06**	Na_3PO_4	10		06	Na_3PO_4	50	0,00768	**
**06**	Na_3PO_4	25		06	Na_3PO_4	50	0,01825	*
**06**	Na_2CO_3	1		06	Na_2CO_3	5	0,46817	Ns
**06**	Na_2CO_3	1		06	Na_2CO_3	10	0,06807	Ns
**06**	Na_2CO_3	1		06	Na_2CO_3	25	0,55525	Ns
**06**	Na_2CO_3	1		06	Na_2CO_3	50	0,14279	Ns
**06**	Na_2CO_3	5		06	Na_2CO_3	10	0,10784	Ns
**06**	Na_2CO_3	5		06	Na_2CO_3	25	0,99059	Ns
**06**	Na_2CO_3	5		06	Na_2CO_3	50	0,29623	Ns
**06**	Na_2CO_3	10		06	Na_2CO_3	25	0,10725	Ns
**06**	Na_2CO_3	10		06	Na_2CO_3	50	0,19259	Ns
**06**	Na_2CO_3	25		06	Na_2CO_3	50	0,41601	Ns
**02**	H2O	1		02	H2O	5	0,00021	***
**02**	H2O	1		02	H2O	10	0,00001	***

**02**	H2O	1	02	H2O	25	0,00029	***
**02**	H2O	1	02	H2O	50	0,00028	***
**02**	H2O	5	02	H2O	10	0,00652	**
**02**	H2O	5	02	H2O	25	0,00503	**
**02**	H2O	5	02	H2O	50	0,00493	**
**02**	H2O	10	02	H2O	25	0,00576	**
**02**	H2O	10	02	H2O	50	0,00540	**
**02**	H2O	25	02	H2O	50	0,13661	Ns
**02**	Na_3PO_4	1	02	Na_3PO_4	5	0,05672	Ns
**02**	Na_3PO_4	1	02	Na_3PO_4	10	0,01151	*
**02**	Na_3PO_4	1	02	Na_3PO_4	25	0,00054	***
**02**	Na_3PO_4	1	02	Na_3PO_4	50	0,12190	Ns
**02**	Na_3PO_4	5	02	Na_3PO_4	10	0,06282	Ns
**02**	Na_3PO_4	5	02	Na_3PO_4	25	0,36463	Ns
**02**	Na_3PO_4	5	02	Na_3PO_4	50	0,97528	Ns
**02**	Na_3PO_4	10	02	Na_3PO_4	25	0,12688	Ns
**02**	Na_3PO_4	10	02	Na_3PO_4	50	0,12688	Ns
**02**	Na_3PO_4	25	02	Na_3PO_4	50	0,53336	Ns
**02**	Na_2CO_3	1	02	Na_2CO_3	5	0,69344	Ns
**02**	Na_2CO_3	1	02	Na_2CO_3	10	0,16638	Ns
**02**	Na_2CO_3	1	02	Na_2CO_3	25	0,09278	Ns
**02**	Na_2CO_3	1	02	Na_2CO_3	50	0,10004	Ns
**02**	Na_2CO_3	5	02	Na_2CO_3	10	0,12487	Ns
**02**	Na_2CO_3	5	02	Na_2CO_3	25	0,07372	Ns
**02**	Na_2CO_3	5	02	Na_2CO_3	50	0,24809	Ns
**02**	Na_2CO_3	10	02	Na_2CO_3	25	0,84472	Ns
**02**	Na_2CO_3	10	02	Na_2CO_3	50	0,04253	*
**02**	Na_2CO_3	25	02	Na_2CO_3	50	0,02045	*
