# Supplementary material for: Interplay of phosphate and carbonate ions with flavin photosensitizers in photodynamic inactivation of bacteria
Source: PLoS One. 2021 Jun 11;16(6):e0253212. doi: 10.1371/journal.pone.0253212 (PMC8195418; doi:10.1371/journal.pone.0253212)
Supplement: S2 File — (PDF) [file pone.0253212.s009.pdf]

Statistical analysis of inactivation data without ions

Method

In order to investigate the gathered data statistically, significance between samples was calculated via unpaired, two-tailed t-tests assuming normal distribution. Events were considered statistically significant for $p < 0.05$ and marked in the following table with one asterisk. When p is < 0.01, events were considered highly significant and marked with two asterisks. Extremely significant events with $p < 0.001$ were marked with three asterisks. Non-significant events were marked with "Ns", whenever a calculation of the corresponding p-value was not possible the values were marked with "Nd".

Results

Table 1: Results of the performed t-tests for each of the listed conditions. The abbreviations represent the following: Sa indicates Staphylococcus aureus. Pa Pseudomonas aeruginosa; DC indicates the dark control, numbers in the same column represent the applied PS concentration in µmol l^{-1}. 06 indicates the PS FLASH-06a while 02 indicates FLASH-02a.

**Condition 1**				**Vs.**	**Condition 2**				**p**	**significance**
**Sa**	06	H_2O	DC		Sa	06	H_2O	0	0.00703	**
**Sa**	06	H_2O	DC		Sa	06	H_2O	1	0.00066	***
**Sa**	06	H_2O	DC		Sa	06	H_2O	5	0.00000	***
**Sa**	06	H_2O	DC		Sa	06	H_2O	10	0.00000	***
**Sa**	06	H_2O	DC		Sa	06	H_2O	25	0.00000	***
**Sa**	06	H_2O	DC		Sa	06	H_2O	50	0.00006	***
**Sa**	06	H_2O	0		Sa	06	H_2O	1	0.00022	***
**Sa**	06	H_2O	0		Sa	06	H_2O	5	0.00001	***
**Sa**	06	H_2O	0		Sa	06	H_2O	10	0.00003	***
**Sa**	06	H_2O	0		Sa	06	H_2O	25	0.00002	***
**Sa**	06	H_2O	0		Sa	06	H_2O	50	0.00038	***
**Sa**	06	H_2O	1		Sa	06	H_2O	5	0.00000	***
**Sa**	06	H_2O	1		Sa	06	H_2O	10	0.00001	***
**Sa**	06	H_2O	1		Sa	06	H_2O	25	0.00001	***
**Sa**	06	H_2O	1		Sa	06	H_2O	50	0.00037	***
**Sa**	06	H_2O	5		Sa	06	H_2O	10	0.00088	***
**Sa**	06	H_2O	5		Sa	06	H_2O	25	0.00005	***
**Sa**	06	H_2O	5		Sa	06	H_2O	50	0.00160	**
**Sa**	06	H_2O	10		Sa	06	H_2O	25	0.00036	***
**Sa**	06	H_2O	10		Sa	06	H_2O	50	0.00262	**
**Sa**	06	H_2O	25		Sa	06	H_2O	50	0.02647	*
**Sa**	02	H_2O	DC		Sa	02	H_2O	0	0.00083	***
**Sa**	02	H_2O	DC		Sa	02	H_2O	1	0.00020	***
**Sa**	02	H_2O	DC		Sa	02	H_2O	5	0.00071	***
**Sa**	02	H_2O	DC		Sa	02	H_2O	10	0.00151	**
**Sa**	02	H_2O	DC		Sa	02	H_2O	25	0.00005	***
**Sa**	02	H_2O	DC		Sa	02	H_2O	50	0.00005	***
**Sa**	02	H_2O	0		Sa	02	H_2O	1	0.00414	**
**Sa**	02	H_2O	0		Sa	02	H_2O	5	0.00179	**
**Sa**	02	H_2O	0		Sa	02	H_2O	10	0.00214	**
**Sa**	02	H_2O	0		Sa	02	H_2O	25	0.00006	***
**Sa**	02	H_2O	0		Sa	02	H_2O	50	0.00006	***

**Sa**	02	H_2O	1	Sa	02	H_2O	5	0.00294	**
**Sa**	02	H_2O	1	Sa	02	H_2O	10	0.00227	**
**Sa**	02	H_2O	1	Sa	02	H_2O	25	0.00012	***
**Sa**	02	H_2O	1	Sa	02	H_2O	50	0.00012	***
**Sa**	02	H_2O	5	Sa	02	H_2O	10	0.00246	**
**Sa**	02	H_2O	5	Sa	02	H_2O	25	0.00065	***
**Sa**	02	H_2O	5	Sa	02	H_2O	50	0.00065	***
**Sa**	02	H_2O	10	Sa	02	H_2O	25	0.00963	**
**Sa**	02	H_2O	10	Sa	02	H_2O	50	0.00963	**
**Sa**	02	H_2O	25	Sa	02	H_2O	50	nd	Nd
**Pa**	06	H_2O	DC	Pa	06	H_2O	0	0.09800	ns
**Pa**	06	H_2O	DC	Pa	06	H_2O	1	0.16111	Ns
**Pa**	06	H_2O	DC	Pa	06	H_2O	5	0.00013	***
**Pa**	06	H_2O	DC	Pa	06	H_2O	10	0.00015	***
**Pa**	06	H_2O	DC	Pa	06	H_2O	25	0.00015	***
**Pa**	06	H_2O	DC	Pa	06	H_2O	50	0.00015	***
**Pa**	06	H_2O	0	Pa	06	H_2O	1	0.82890	Ns
**Pa**	06	H_2O	0	Pa	06	H_2O	5	0.00009	***
**Pa**	06	H_2O	0	Pa	06	H_2O	10	0.00010	***
**Pa**	06	H_2O	0	Pa	06	H_2O	25	0.00010	***
**Pa**	06	H_2O	0	Pa	06	H_2O	50	0.00010	***
**Pa**	06	H_2O	1	Pa	06	H_2O	5	0.00028	***
**Pa**	06	H_2O	1	Pa	06	H_2O	10	0.00017	***
**Pa**	06	H_2O	1	Pa	06	H_2O	25	0.00017	***
**Pa**	06	H_2O	1	Pa	06	H_2O	50	0.00017	***
**Pa**	06	H_2O	5	Pa	06	H_2O	10	0.00015	***
**Pa**	06	H_2O	5	Pa	06	H_2O	25	0.00015	***
**Pa**	06	H_2O	5	Pa	06	H_2O	50	0.00015	***
**Pa**	06	H_2O	10	Pa	06	H_2O	25	Nd	Nd
**Pa**	06	H_2O	10	Pa	06	H_2O	50	Nd	Nd
**Pa**	06	H_2O	25	Pa	06	H_2O	50	Nd	Nd
**Pa**	02	H_2O	DC	Pa	02	H_2O	0	0.00023	***
**Pa**	02	H_2O	DC	Pa	02	H_2O	1	0.00155	**
**Pa**	02	H_2O	DC	Pa	02	H_2O	5	0.00293	**
**Pa**	02	H_2O	DC	Pa	02	H_2O	10	0.00001	***
**Pa**	02	H_2O	DC	Pa	02	H_2O	25	0.00001	***
**Pa**	02	H_2O	DC	Pa	02	H_2O	50	0.00001	***
**Pa**	02	H_2O	0	Pa	02	H_2O	1	0.00860	**
**Pa**	02	H_2O	0	Pa	02	H_2O	5	0.00383	**
**Pa**	02	H_2O	0	Pa	02	H_2O	10	0.00003	***
**Pa**	02	H_2O	0	Pa	02	H_2O	25	0.00003	***
**Pa**	02	H_2O	0	Pa	02	H_2O	50	0.00003	***
**Pa**	02	H_2O	1	Pa	02	H_2O	5	0.00361	**
**Pa**	02	H_2O	1	Pa	02	H_2O	10	0.00012	***
**Pa**	02	H_2O	1	Pa	02	H_2O	25	0.00012	***
**Pa**	02	H_2O	1	Pa	02	H_2O	50	0.00012	***
**Pa**	02	H_2O	5	Pa	02	H_2O	10	0.00790	**
**Pa**	02	H_2O	5	Pa	02	H_2O	25	0.00790	**
**Pa**	02	H_2O	5	Pa	02	H_2O	50	0.00790	**

**Pa**	02	H_2O	10	Pa	02	H_2O	25	Nd	Nd
**Pa**	02	H_2O	10	Pa	02	H_2O	50	Nd	Nd
**Pa**	02	H_2O	25	Pa	02	H_2O	50	nd	Nd
**Sa**	06	H_2O	1	Sa	02	H_2O	1	0.01466	*
**Sa**	06	H_2O	1	Pa	06	H_2O	1	0.01079	*
**Sa**	02	H_2O	1	Pa	02	H_2O	1	0.06015	*
**Pa**	06	H_2O	1	Pa	02	H_2O	1	0.00157	**
**Sa**	06	H_2O	5	Sa	02	H_2O	5	0.04187	*
**Sa**	06	H_2O	5	Pa	06	H_2O	5	0.00057	***
**Sa**	02	H_2O	5	Pa	02	H_2O	5	0.00769	**
**Pa**	06	H_2O	5	Pa	02	H_2O	5	0.00569	**
**Sa**	06	H_2O	10	Sa	02	H_2O	10	0.02294	*
**Sa**	06	H_2O	10	Pa	06	H_2O	10	0.00012	***
**Sa**	02	H_2O	10	Pa	02	H_2O	10	0.00963	**
**Sa**	06	H_2O	25	Sa	02	H_2O	25	0.00018	***
**Sa**	06	H_2O	25	Pa	06	H_2O	25	0.00018	***
**Sa**	06	H_2O	50	Sa	02	H_2O	25	0.00129	**
**Sa**	06	H_2O	50	Pa	06	H_2O	50	0.00129	**
