# Supplementary material for: Interplay of phosphate and carbonate ions with flavin photosensitizers in photodynamic inactivation of bacteria
Source: PLoS One. 2021 Jun 11;16(6):e0253212. doi: 10.1371/journal.pone.0253212 (PMC8195418; doi:10.1371/journal.pone.0253212)
Supplement: S3 File — (PDF) [file pone.0253212.s010.pdf]

Statistical analysis of *Staphylococcus aureus* inactivation data

Method

In order to investigate the gathered data statistically, significance between samples was calculated via unpaired, two-tailed t-tests assuming normal distribution. Events were considered statistically significant for $p < 0.05$ and marked in the following table with one asterisk. When p is < 0.01, events were considered highly significant and marked with two asterisks. Extremely significant events with $p < 0.001$ were marked with three asterisks. Non-significant events were marked with "Ns", whenever a calculation of the corresponding p-value was not possible the values were marked with "Nd".

Results

Table 1: Results of the performed t-tests for each of the listed conditions. The abbreviations represent the following: DC indicates the dark control, numbers in the same column represent the applied PS concentration in µmol l^{-1}. 06 indicates the PS FLASH-06a while 02 indicates FLASH-02a. Applied salts are abbreviated as empirical formulas with their respective concentration in the next column.

**Condition 1**				**Vs.**	**Condition 2**				**p**	**significance**
**06**	DC	Na_2CO_3	75		06	0	Na_2CO_3	75	0.03281	*
**06**	DC	Na_2CO_3	75		06	1	Na_2CO_3	75	0.00093	***
**06**	DC	Na_2CO_3	75		06	5	Na_2CO_3	75	0.00046	***
**06**	DC	Na_2CO_3	75		06	10	Na_2CO_3	75	0.00098	***
**06**	DC	Na_2CO_3	75		06	25	Na_2CO_3	75	0.00020	***
**06**	DC	Na_2CO_3	75		06	50	Na_2CO_3	75	0.00001	***
**06**	0	Na_2CO_3	75		06	1	Na_2CO_3	75	0.24805	Ns
**06**	0	Na_2CO_3	75		06	5	Na_2CO_3	75	0.04645	*
**06**	0	Na_2CO_3	75		06	10	Na_2CO_3	75	0.01986	*
**06**	0	Na_2CO_3	75		06	25	Na_2CO_3	75	0.07577	Ns
**06**	0	Na_2CO_3	75		06	50	Na_2CO_3	75	0.00182	**
**06**	1	Na_2CO_3	75		06	5	Na_2CO_3	75	0.04588	*
**06**	1	Na_2CO_3	75		06	10	Na_2CO_3	75	0.01818	*
**06**	1	Na_2CO_3	75		06	25	Na_2CO_3	75	0.06215	Ns
**06**	1	Na_2CO_3	75		06	50	Na_2CO_3	75	0.00007	***
**06**	5	Na_2CO_3	75		06	10	Na_2CO_3	75	0.22057	Ns
**06**	5	Na_2CO_3	75		06	25	Na_2CO_3	75	0.55600	Ns
**06**	5	Na_2CO_3	75		06	50	Na_2CO_3	75	0.00018	***
**06**	10	Na_2CO_3	75		06	25	Na_2CO_3	75	0.12442	Ns
**06**	10	Na_2CO_3	75		06	50	Na_2CO_3	75	0.00085	***
**06**	25	Na_2CO_3	75		06	50	Na_2CO_3	75	0.00040	***
**06**	DC	Na_2CO_3	7.5		06	0	Na_2CO_3	7.5	0.05597	Ns
**06**	DC	Na_2CO_3	7.5		06	1	Na_2CO_3	7.5	0.15116	Ns
**06**	DC	Na_2CO_3	7.5		06	5	Na_2CO_3	7.5	0.27780	Ns
**06**	DC	Na_2CO_3	7.5		06	10	Na_2CO_3	7.5	0.51916	Ns
**06**	DC	Na_2CO_3	7.5		06	25	Na_2CO_3	7.5	0.63046	Ns
**06**	DC	Na_2CO_3	7.5		06	50	Na_2CO_3	7.5	0.90338	Ns
**06**	0	Na_2CO_3	7.5		06	1	Na_2CO_3	7.5	0.31870	Ns
**06**	0	Na_2CO_3	7.5		06	5	Na_2CO_3	7.5	0.17129	Ns
**06**	0	Na_2CO_3	7.5		06	10	Na_2CO_3	7.5	0.09152	Ns
**06**	0	Na_2CO_3	7.5		06	25	Na_2CO_3	7.5	0.06716	Ns
**06**	0	Na_2CO_3	7.5		06	50	Na_2CO_3	7.5	0.05435	Ns

**06**	1	Na_2CO_3	7.5	06	5	Na_2CO_3	7.5	0.58203	Ns
**06**	1	Na_2CO_3	7.5	06	10	Na_2CO_3	7.5	0.29211	Ns
**06**	1	Na_2CO_3	7.5	06	25	Na_2CO_3	7.5	0.20424	Ns
**06**	1	Na_2CO_3	7.5	06	50	Na_2CO_3	7.5	0.15253	Ns
**06**	5	Na_2CO_3	7.5	06	10	Na_2CO_3	7.5	0.57108	Ns
**06**	5	Na_2CO_3	7.5	06	25	Na_2CO_3	7.5	0.42595	Ns
**06**	5	Na_2CO_3	7.5	06	50	Na_2CO_3	7.5	0.29631	Ns
**06**	10	Na_2CO_3	7.5	06	25	Na_2CO_3	7.5	0.82440	Ns
**06**	10	Na_2CO_3	7.5	06	50	Na_2CO_3	7.5	0.57497	Ns
**06**	25	Na_2CO_3	7.5	06	50	Na_2CO_3	7.5	0.70336	Ns
**06**	DC	Na_2CO_3	0.75	06	0	Na_2CO_3	0.75	0.01831	*
**06**	DC	Na_2CO_3	0.75	06	1	Na_2CO_3	0.75	0.10782	Ns
**06**	DC	Na_2CO_3	0.75	06	5	Na_2CO_3	0.75	0.04161	*
**06**	DC	Na_2CO_3	0.75	06	10	Na_2CO_3	0.75	0.00191	**
**06**	DC	Na_2CO_3	0.75	06	25	Na_2CO_3	0.75	0.00577	**
**06**	DC	Na_2CO_3	0.75	06	50	Na_2CO_3	0.75	0.00620	**
**06**	0	Na_2CO_3	0.75	06	1	Na_2CO_3	0.75	0.09681	Ns
**06**	0	Na_2CO_3	0.75	06	5	Na_2CO_3	0.75	0.00095	***
**06**	0	Na_2CO_3	0.75	06	10	Na_2CO_3	0.75	0.00295	**
**06**	0	Na_2CO_3	0.75	06	25	Na_2CO_3	0.75	0.00559	**
**06**	0	Na_2CO_3	0.75	06	50	Na_2CO_3	0.75	0.00597	**
**06**	1	Na_2CO_3	0.75	06	5	Na_2CO_3	0.75	0.01260	*
**06**	1	Na_2CO_3	0.75	06	10	Na_2CO_3	0.75	0.00168	**
**06**	1	Na_2CO_3	0.75	06	25	Na_2CO_3	0.75	0.00556	**
**06**	1	Na_2CO_3	0.75	06	50	Na_2CO_3	0.75	0.00597	**
**06**	5	Na_2CO_3	0.75	06	10	Na_2CO_3	0.75	0.00894	**
**06**	5	Na_2CO_3	0.75	06	25	Na_2CO_3	0.75	0.00676	**
**06**	5	Na_2CO_3	0.75	06	50	Na_2CO_3	0.75	0.00714	**
**06**	10	Na_2CO_3	0.75	06	25	Na_2CO_3	0.75	0.00784	**
**06**	10	Na_2CO_3	0.75	06	50	Na_2CO_3	0.75	0.00844	**
**06**	25	Na_2CO_3	0.75	06	50	Na_2CO_3	0.75	0.69866	Ns
**06**	DC	Na_2CO_3	0.075	06	0	Na_2CO_3	0.075	0.43317	Ns
**06**	DC	Na_2CO_3	0.075	06	1	Na_2CO_3	0.075	0.02347	*
**06**	DC	Na_2CO_3	0.075	06	5	Na_2CO_3	0.075	0.00084	***
**06**	DC	Na_2CO_3	0.075	06	10	Na_2CO_3	0.075	0.00159	**
**06**	DC	Na_2CO_3	0.075	06	25	Na_2CO_3	0.075	0.00123	**
**06**	DC	Na_2CO_3	0.075	06	50	Na_2CO_3	0.075	0.00113	**
**06**	0	Na_2CO_3	0.075	06	1	Na_2CO_3	0.075	0.08280	Ns
**06**	0	Na_2CO_3	0.075	06	5	Na_2CO_3	0.075	0.00066	***
**06**	0	Na_2CO_3	0.075	06	10	Na_2CO_3	0.075	0.00152	**
**06**	0	Na_2CO_3	0.075	06	25	Na_2CO_3	0.075	0.00116	**
**06**	0	Na_2CO_3	0.075	06	50	Na_2CO_3	0.075	0.00106	**
**06**	1	Na_2CO_3	0.075	06	5	Na_2CO_3	0.075	0.00088	***
**06**	1	Na_2CO_3	0.075	06	10	Na_2CO_3	0.075	0.00164	**
**06**	1	Na_2CO_3	0.075	06	25	Na_2CO_3	0.075	0.00128	**
**06**	1	Na_2CO_3	0.075	06	50	Na_2CO_3	0.075	0.00116	**
**06**	5	Na_2CO_3	0.075	06	10	Na_2CO_3	0.075	0.00221	**
**06**	5	Na_2CO_3	0.075	06	25	Na_2CO_3	0.075	0.00148	**
**06**	5	Na_2CO_3	0.075	06	50	Na_2CO_3	0.075	0.00108	**
**06**	10	Na_2CO_3	0.075	06	25	Na_2CO_3	0.075	0.20364	Ns
**06**	10	Na_2CO_3	0.075	06	50	Na_2CO_3	0.075	0.98838	Ns

**06**	25	Na_2CO_3	0.075	06	50	Na_2CO_3	0.075	0.17553	Ns
**06**	DC	Na_3PO_4	75	06	0	Na_3PO_4	75	0.07447	Ns
**06**	DC	Na_3PO_4	75	06	1	Na_3PO_4	75	0.02987	*
**06**	DC	Na_3PO_4	75	06	5	Na_3PO_4	75	0.18448	Ns
**06**	DC	Na_3PO_4	75	06	10	Na_3PO_4	75	0.00525	**
**06**	DC	Na_3PO_4	75	06	25	Na_3PO_4	75	0.00901	**
**06**	DC	Na_3PO_4	75	06	50	Na_3PO_4	75	0.00016	***
**06**	0	Na_3PO_4	75	06	1	Na_3PO_4	75	0.67574	Ns
**06**	0	Na_3PO_4	75	06	5	Na_3PO_4	75	0.16120	Ns
**06**	0	Na_3PO_4	75	06	10	Na_3PO_4	75	0.47191	Ns
**06**	0	Na_3PO_4	75	06	25	Na_3PO_4	75	0.90854	Ns
**06**	0	Na_3PO_4	75	06	50	Na_3PO_4	75	0.00136	**
**06**	1	Na_3PO_4	75	06	5	Na_3PO_4	75	0.09336	Ns
**06**	1	Na_3PO_4	75	06	10	Na_3PO_4	75	0.13703	Ns
**06**	1	Na_3PO_4	75	06	25	Na_3PO_4	75	0.37851	Ns
**06**	1	Na_3PO_4	75	06	50	Na_3PO_4	75	0.00024	***
**06**	5	Na_3PO_4	75	06	10	Na_3PO_4	75	0.01495	*
**06**	5	Na_3PO_4	75	06	25	Na_3PO_4	75	0.00386	**
**06**	5	Na_3PO_4	75	06	50	Na_3PO_4	75	0.00088	***
**06**	10	Na_3PO_4	75	06	25	Na_3PO_4	75	0.27389	Ns
**06**	10	Na_3PO_4	75	06	50	Na_3PO_4	75	0.00043	***
**06**	25	Na_3PO_4	75	06	50	Na_3PO_4	75	0.00142	**
**06**	DC	Na_3PO_4	7.5	06	0	Na_3PO_4	7.5	0.00060	***
**06**	DC	Na_3PO_4	7.5	06	1	Na_3PO_4	7.5	0.00116	**
**06**	DC	Na_3PO_4	7.5	06	5	Na_3PO_4	7.5	0.00077	***
**06**	DC	Na_3PO_4	7.5	06	10	Na_3PO_4	7.5	0.00148	**
**06**	DC	Na_3PO_4	7.5	06	25	Na_3PO_4	7.5	0.00082	***
**06**	DC	Na_3PO_4	7.5	06	50	Na_3PO_4	7.5	0.01273	*
**06**	0	Na_3PO_4	7.5	06	1	Na_3PO_4	7.5	0.16605	Ns
**06**	0	Na_3PO_4	7.5	06	5	Na_3PO_4	7.5	0.82641	Ns
**06**	0	Na_3PO_4	7.5	06	10	Na_3PO_4	7.5	0.05925	Ns
**06**	0	Na_3PO_4	7.5	06	25	Na_3PO_4	7.5	0.25671	Ns
**06**	0	Na_3PO_4	7.5	06	50	Na_3PO_4	7.5	0.00035	***
**06**	1	Na_3PO_4	7.5	06	5	Na_3PO_4	7.5	0.15684	Ns
**06**	1	Na_3PO_4	7.5	06	10	Na_3PO_4	7.5	0.46593	Ns
**06**	1	Na_3PO_4	7.5	06	25	Na_3PO_4	7.5	0.60142	Ns
**06**	1	Na_3PO_4	7.5	06	50	Na_3PO_4	7.5	0.00044	***
**06**	5	Na_3PO_4	7.5	06	10	Na_3PO_4	7.5	0.03030	*
**06**	5	Na_3PO_4	7.5	06	25	Na_3PO_4	7.5	0.22093	Ns
**06**	5	Na_3PO_4	7.5	06	50	Na_3PO_4	7.5	0.00002	***
**06**	10	Na_3PO_4	7.5	06	25	Na_3PO_4	7.5	0.17486	Ns
**06**	10	Na_3PO_4	7.5	06	50	Na_3PO_4	7.5	0.00008	***
**06**	25	Na_3PO_4	7.5	06	50	Na_3PO_4	7.5	0.00007	***
**06**	DC	Na_3PO_4	0.75	06	0	Na_3PO_4	0.75	0.45247	Ns
**06**	DC	Na_3PO_4	0.75	06	1	Na_3PO_4	0.75	0.29622	Ns
**06**	DC	Na_3PO_4	0.75	06	5	Na_3PO_4	0.75	0.00040	***
**06**	DC	Na_3PO_4	0.75	06	10	Na_3PO_4	0.75	0.00032	***
**06**	DC	Na_3PO_4	0.75	06	25	Na_3PO_4	0.75	0.00025	***
**06**	DC	Na_3PO_4	0.75	06	50	Na_3PO_4	0.75	0.00033	***
**06**	0	Na_3PO_4	0.75	06	1	Na_3PO_4	0.75	0.87929	Ns
**06**	0	Na_3PO_4	0.75	06	5	Na_3PO_4	0.75	0.00005	***

**06**	0	Na_3PO_4	0.75	06	10	Na_3PO_4	0.75	0.00007	***
**06**	0	Na_3PO_4	0.75	06	25	Na_3PO_4	0.75	0.00003	***
**06**	0	Na_3PO_4	0.75	06	50	Na_3PO_4	0.75	0.00004	***
**06**	1	Na_3PO_4	0.75	06	5	Na_3PO_4	0.75	0.00010	***
**06**	1	Na_3PO_4	0.75	06	10	Na_3PO_4	0.75	0.00012	***
**06**	1	Na_3PO_4	0.75	06	25	Na_3PO_4	0.75	0.00006	***
**06**	1	Na_3PO_4	0.75	06	50	Na_3PO_4	0.75	0.00008	***
**06**	5	Na_3PO_4	0.75	06	10	Na_3PO_4	0.75	0.00040	***
**06**	5	Na_3PO_4	0.75	06	25	Na_3PO_4	0.75	0.00209	**
**06**	5	Na_3PO_4	0.75	06	50	Na_3PO_4	0.75	0.06352	Ns
**06**	10	Na_3PO_4	0.75	06	25	Na_3PO_4	0.75	0.00742	**
**06**	10	Na_3PO_4	0.75	06	50	Na_3PO_4	0.75	0.00092	***
**06**	25	Na_3PO_4	0.75	06	50	Na_3PO_4	0.75	0.01050	*
**06**	DC	Na_3PO_4	0.075	06	0	Na_3PO_4	0.075	0.01025	*
**06**	DC	Na_3PO_4	0.075	06	1	Na_3PO_4	0.075	0.00236	**
**06**	DC	Na_3PO_4	0.075	06	5	Na_3PO_4	0.075	0.00033	***
**06**	DC	Na_3PO_4	0.075	06	10	Na_3PO_4	0.075	0.00376	**
**06**	DC	Na_3PO_4	0.075	06	25	Na_3PO_4	0.075	0.00001	***
**06**	DC	Na_3PO_4	0.075	06	50	Na_3PO_4	0.075	0.00001	***
**06**	0	Na_3PO_4	0.075	06	1	Na_3PO_4	0.075	0.47051	Ns
**06**	0	Na_3PO_4	0.075	06	5	Na_3PO_4	0.075	0.00026	***
**06**	0	Na_3PO_4	0.075	06	10	Na_3PO_4	0.075	0.00328	**
**06**	0	Na_3PO_4	0.075	06	25	Na_3PO_4	0.075	0.00005	***
**06**	0	Na_3PO_4	0.075	06	50	Na_3PO_4	0.075	0.00005	***
**06**	1	Na_3PO_4	0.075	06	5	Na_3PO_4	0.075	0.00018	***
**06**	1	Na_3PO_4	0.075	06	10	Na_3PO_4	0.075	0.00419	**
**06**	1	Na_3PO_4	0.075	06	25	Na_3PO_4	0.075	0.00002	***
**06**	1	Na_3PO_4	0.075	06	50	Na_3PO_4	0.075	0.00002	***
**06**	5	Na_3PO_4	0.075	06	10	Na_3PO_4	0.075	0.00877	**
**06**	5	Na_3PO_4	0.075	06	25	Na_3PO_4	0.075	0.00006	***
**06**	5	Na_3PO_4	0.075	06	50	Na_3PO_4	0.075	0.00006	***
**06**	10	Na_3PO_4	0.075	06	25	Na_3PO_4	0.075	0.00176	**
**06**	10	Na_3PO_4	0.075	06	50	Na_3PO_4	0.075	0.00176	**
**06**	25	Na_3PO_4	0.075	06	50	Na_3PO_4	0.075	Nd	Nd
**02**	DC	Na_2CO_3	75	02	0	Na_2CO_3	75	0.01831	*
**02**	DC	Na_2CO_3	75	02	1	Na_2CO_3	75	0.00379	**
**02**	DC	Na_2CO_3	75	02	5	Na_2CO_3	75	0.00235	**
**02**	DC	Na_2CO_3	75	02	10	Na_2CO_3	75	0.00300	**
**02**	DC	Na_2CO_3	75	02	25	Na_2CO_3	75	0.00042	***
**02**	DC	Na_2CO_3	75	02	50	Na_2CO_3	75	0.00132	**
**02**	0	Na_2CO_3	75	02	1	Na_2CO_3	75	0.11601	Ns
**02**	0	Na_2CO_3	75	02	5	Na_2CO_3	75	0.06644	Ns
**02**	0	Na_2CO_3	75	02	10	Na_2CO_3	75	0.05084	Ns
**02**	0	Na_2CO_3	75	02	25	Na_2CO_3	75	0.03549	*
**02**	0	Na_2CO_3	75	02	50	Na_2CO_3	75	0.01096	*
**02**	1	Na_2CO_3	75	02	5	Na_2CO_3	75	0.65101	Ns
**02**	1	Na_2CO_3	75	02	10	Na_2CO_3	75	0.45546	Ns
**02**	1	Na_2CO_3	75	02	25	Na_2CO_3	75	0.31903	Ns
**02**	1	Na_2CO_3	75	02	50	Na_2CO_3	75	0.05944	Ns
**02**	5	Na_2CO_3	75	02	10	Na_2CO_3	75	0.73176	Ns
**02**	5	Na_2CO_3	75	02	25	Na_2CO_3	75	0.57051	Ns

**02**	5	Na_2CO_3	75	02	50	Na_2CO_3	75	0.09376	Ns
**02**	10	Na_2CO_3	75	02	25	Na_2CO_3	75	0.87657	Ns
**02**	10	Na_2CO_3	75	02	50	Na_2CO_3	75	0.15645	Ns
**02**	25	Na_2CO_3	75	02	50	Na_2CO_3	75	0.14017	Ns
**02**	DC	Na_2CO_3	7.5	02	0	Na_2CO_3	7.5	0.02561	*
**02**	DC	Na_2CO_3	7.5	02	1	Na_2CO_3	7.5	0.00514	**
**02**	DC	Na_2CO_3	7.5	02	5	Na_2CO_3	7.5	0.00615	**
**02**	DC	Na_2CO_3	7.5	02	10	Na_2CO_3	7.5	0.00418	**
**02**	DC	Na_2CO_3	7.5	02	25	Na_2CO_3	7.5	0.00461	**
**02**	DC	Na_2CO_3	7.5	02	50	Na_2CO_3	7.5	0.00413	**
**02**	0	Na_2CO_3	7.5	02	1	Na_2CO_3	7.5	0.14893	Ns
**02**	0	Na_2CO_3	7.5	02	5	Na_2CO_3	7.5	0.05647	Ns
**02**	0	Na_2CO_3	7.5	02	10	Na_2CO_3	7.5	0.03657	*
**02**	0	Na_2CO_3	7.5	02	25	Na_2CO_3	7.5	0.00846	**
**02**	0	Na_2CO_3	7.5	02	50	Na_2CO_3	7.5	0.00626	**
**02**	1	Na_2CO_3	7.5	02	5	Na_2CO_3	7.5	0.33291	Ns
**02**	1	Na_2CO_3	7.5	02	10	Na_2CO_3	7.5	0.18990	Ns
**02**	1	Na_2CO_3	7.5	02	25	Na_2CO_3	7.5	0.02569	*
**02**	1	Na_2CO_3	7.5	02	50	Na_2CO_3	7.5	0.01754	*
**02**	5	Na_2CO_3	7.5	02	10	Na_2CO_3	7.5	0.71522	Ns
**02**	5	Na_2CO_3	7.5	02	25	Na_2CO_3	7.5	0.07168	Ns
**02**	5	Na_2CO_3	7.5	02	50	Na_2CO_3	7.5	0.04516	*
**02**	10	Na_2CO_3	7.5	02	25	Na_2CO_3	7.5	0.09912	Ns
**02**	10	Na_2CO_3	7.5	02	50	Na_2CO_3	7.5	0.06074	Ns
**02**	25	Na_2CO_3	7.5	02	50	Na_2CO_3	7.5	0.69508	Ns
**02**	DC	Na_2CO_3	0.75	02	0	Na_2CO_3	0.75	0.02495	*
**02**	DC	Na_2CO_3	0.75	02	1	Na_2CO_3	0.75	0.00054	***
**02**	DC	Na_2CO_3	0.75	02	5	Na_2CO_3	0.75	0.00093	***
**02**	DC	Na_2CO_3	0.75	02	10	Na_2CO_3	0.75	0.00148	**
**02**	DC	Na_2CO_3	0.75	02	25	Na_2CO_3	0.75	0.00097	***
**02**	DC	Na_2CO_3	0.75	02	50	Na_2CO_3	0.75	0.00196	**
**02**	0	Na_2CO_3	0.75	02	1	Na_2CO_3	0.75	0.00153	**
**02**	0	Na_2CO_3	0.75	02	5	Na_2CO_3	0.75	0.00041	***
**02**	0	Na_2CO_3	0.75	02	10	Na_2CO_3	0.75	0.00095	***
**02**	0	Na_2CO_3	0.75	02	25	Na_2CO_3	0.75	0.00055	***
**02**	0	Na_2CO_3	0.75	02	50	Na_2CO_3	0.75	0.00177	**
**02**	1	Na_2CO_3	0.75	02	5	Na_2CO_3	0.75	0.00116	**
**02**	1	Na_2CO_3	0.75	02	10	Na_2CO_3	0.75	0.00168	**
**02**	1	Na_2CO_3	0.75	02	25	Na_2CO_3	0.75	0.00090	***
**02**	1	Na_2CO_3	0.75	02	50	Na_2CO_3	0.75	0.00224	**
**02**	5	Na_2CO_3	0.75	02	10	Na_2CO_3	0.75	0.00948	**
**02**	5	Na_2CO_3	0.75	02	25	Na_2CO_3	0.75	0.00200	**
**02**	5	Na_2CO_3	0.75	02	50	Na_2CO_3	0.75	0.00247	**
**02**	10	Na_2CO_3	0.75	02	25	Na_2CO_3	0.75	0.07958	Ns
**02**	10	Na_2CO_3	0.75	02	50	Na_2CO_3	0.75	0.00291	**
**02**	25	Na_2CO_3	0.75	02	50	Na_2CO_3	0.75	0.00533	**
**02**	DC	Na_2CO_3	0.075	02	0	Na_2CO_3	0.075	0.11861	Ns
**02**	DC	Na_2CO_3	0.075	02	1	Na_2CO_3	0.075	0.00137	**
**02**	DC	Na_2CO_3	0.075	02	5	Na_2CO_3	0.075	0.00138	**
**02**	DC	Na_2CO_3	0.075	02	10	Na_2CO_3	0.075	0.00312	**
**02**	DC	Na_2CO_3	0.075	02	25	Na_2CO_3	0.075	0.00430	**

**02**	DC	Na_2CO_3	0.075	02	50	Na_2CO_3	0.075	0.00001	***
**02**	0	Na_2CO_3	0.075	02	1	Na_2CO_3	0.075	0.00163	**
**02**	0	Na_2CO_3	0.075	02	5	Na_2CO_3	0.075	0.00154	**
**02**	0	Na_2CO_3	0.075	02	10	Na_2CO_3	0.075	0.00197	**
**02**	0	Na_2CO_3	0.075	02	25	Na_2CO_3	0.075	0.00384	**
**02**	0	Na_2CO_3	0.075	02	50	Na_2CO_3	0.075	0.00005	***
**02**	1	Na_2CO_3	0.075	02	5	Na_2CO_3	0.075	0.89925	Ns
**02**	1	Na_2CO_3	0.075	02	10	Na_2CO_3	0.075	0.00371	**
**02**	1	Na_2CO_3	0.075	02	25	Na_2CO_3	0.075	0.00523	**
**02**	1	Na_2CO_3	0.075	02	50	Na_2CO_3	0.075	0.00004	***
**02**	5	Na_2CO_3	0.075	02	10	Na_2CO_3	0.075	0.00372	**
**02**	5	Na_2CO_3	0.075	02	25	Na_2CO_3	0.075	0.00524	**
**02**	5	Na_2CO_3	0.075	02	50	Na_2CO_3	0.075	0.00004	***
**02**	10	Na_2CO_3	0.075	02	25	Na_2CO_3	0.075	0.01531	*
**02**	10	Na_2CO_3	0.075	02	50	Na_2CO_3	0.075	0.00129	**
**02**	25	Na_2CO_3	0.075	02	50	Na_2CO_3	0.075	0.00997	**
**02**	DC	Na_3PO_4	75	02	0	Na_3PO_4	75	0.26025	Ns
**02**	DC	Na_3PO_4	75	02	1	Na_3PO_4	75	0.20908	Ns
**02**	DC	Na_3PO_4	75	02	5	Na_3PO_4	75	0.18274	Ns
**02**	DC	Na_3PO_4	75	02	10	Na_3PO_4	75	0.14970	Ns
**02**	DC	Na_3PO_4	75	02	25	Na_3PO_4	75	0.24606	Ns
**02**	DC	Na_3PO_4	75	02	50	Na_3PO_4	75	0.13981	Ns
**02**	0	Na_3PO_4	75	02	1	Na_3PO_4	75	0.56179	Ns
**02**	0	Na_3PO_4	75	02	5	Na_3PO_4	75	0.54724	Ns
**02**	0	Na_3PO_4	75	02	10	Na_3PO_4	75	0.44302	Ns
**02**	0	Na_3PO_4	75	02	25	Na_3PO_4	75	0.66927	Ns
**02**	0	Na_3PO_4	75	02	50	Na_3PO_4	75	0.38580	Ns
**02**	1	Na_3PO_4	75	02	5	Na_3PO_4	75	0.92823	Ns
**02**	1	Na_3PO_4	75	02	10	Na_3PO_4	75	1.00000	Ns
**02**	1	Na_3PO_4	75	02	25	Na_3PO_4	75	0.89114	Ns
**02**	1	Na_3PO_4	75	02	50	Na_3PO_4	75	0.94735	Ns
**02**	5	Na_3PO_4	75	02	10	Na_3PO_4	75	0.91455	Ns
**02**	5	Na_3PO_4	75	02	25	Na_3PO_4	75	0.95147	Ns
**02**	5	Na_3PO_4	75	02	50	Na_3PO_4	75	0.96839	Ns
**02**	10	Na_3PO_4	75	02	25	Na_3PO_4	75	0.87587	Ns
**02**	10	Na_3PO_4	75	02	50	Na_3PO_4	75	0.93326	Ns
**02**	25	Na_3PO_4	75	02	50	Na_3PO_4	75	0.91982	Ns
**02**	DC	Na_3PO_4	7.5	02	0	Na_3PO_4	7.5	0.02386	*
**02**	DC	Na_3PO_4	7.5	02	1	Na_3PO_4	7.5	0.00700	**
**02**	DC	Na_3PO_4	7.5	02	5	Na_3PO_4	7.5	0.00720	**
**02**	DC	Na_3PO_4	7.5	02	10	Na_3PO_4	7.5	0.00988	**
**02**	DC	Na_3PO_4	7.5	02	25	Na_3PO_4	7.5	0.00070	***
**02**	DC	Na_3PO_4	7.5	02	50	Na_3PO_4	7.5	0.00004	***
**02**	0	Na_3PO_4	7.5	02	1	Na_3PO_4	7.5	0.36053	Ns
**02**	0	Na_3PO_4	7.5	02	5	Na_3PO_4	7.5	0.28356	Ns
**02**	0	Na_3PO_4	7.5	02	10	Na_3PO_4	7.5	0.74684	Ns
**02**	0	Na_3PO_4	7.5	02	25	Na_3PO_4	7.5	0.69795	Ns
**02**	0	Na_3PO_4	7.5	02	50	Na_3PO_4	7.5	0.12815	Ns
**02**	1	Na_3PO_4	7.5	02	5	Na_3PO_4	7.5	0.74770	Ns
**02**	1	Na_3PO_4	7.5	02	10	Na_3PO_4	7.5	0.40004	Ns
**02**	1	Na_3PO_4	7.5	02	25	Na_3PO_4	7.5	0.30174	Ns

**02**	1	Na_3PO_4	7.5	02	50	Na_3PO_4	7.5	0.00884	**
**02**	5	Na_3PO_4	7.5	02	10	Na_3PO_4	7.5	0.28040	Ns
**02**	5	Na_3PO_4	7.5	02	25	Na_3PO_4	7.5	0.18432	Ns
**02**	5	Na_3PO_4	7.5	02	50	Na_3PO_4	7.5	0.00651	**
**02**	10	Na_3PO_4	7.5	02	25	Na_3PO_4	7.5	0.95833	Ns
**02**	10	Na_3PO_4	7.5	02	50	Na_3PO_4	7.5	0.03554	*
**02**	25	Na_3PO_4	7.5	02	50	Na_3PO_4	7.5	0.00291	**
**02**	DC	Na_3PO_4	0.75	02	0	Na_3PO_4	0.75	0.00519	**
**02**	DC	Na_3PO_4	0.75	02	1	Na_3PO_4	0.75	0.00450	**
**02**	DC	Na_3PO_4	0.75	02	5	Na_3PO_4	0.75	0.00151	**
**02**	DC	Na_3PO_4	0.75	02	10	Na_3PO_4	0.75	0.00209	**
**02**	DC	Na_3PO_4	0.75	02	25	Na_3PO_4	0.75	0.00155	**
**02**	DC	Na_3PO_4	0.75	02	50	Na_3PO_4	0.75	0.00001	***
**02**	0	Na_3PO_4	0.75	02	1	Na_3PO_4	0.75	0.07142	Ns
**02**	0	Na_3PO_4	0.75	02	5	Na_3PO_4	0.75	0.00052	***
**02**	0	Na_3PO_4	0.75	02	10	Na_3PO_4	0.75	0.00111	**
**02**	0	Na_3PO_4	0.75	02	25	Na_3PO_4	0.75	0.00069	***
**02**	0	Na_3PO_4	0.75	02	50	Na_3PO_4	0.75	0.00018	***
**02**	1	Na_3PO_4	0.75	02	5	Na_3PO_4	0.75	0.00052	***
**02**	1	Na_3PO_4	0.75	02	10	Na_3PO_4	0.75	0.00108	**
**02**	1	Na_3PO_4	0.75	02	25	Na_3PO_4	0.75	0.00059	***
**02**	1	Na_3PO_4	0.75	02	50	Na_3PO_4	0.75	0.00027	***
**02**	5	Na_3PO_4	0.75	02	10	Na_3PO_4	0.75	0.52359	Ns
**02**	5	Na_3PO_4	0.75	02	25	Na_3PO_4	0.75	0.01865	*
**02**	5	Na_3PO_4	0.75	02	50	Na_3PO_4	0.75	0.00169	**
**02**	10	Na_3PO_4	0.75	02	25	Na_3PO_4	0.75	0.03979	*
**02**	10	Na_3PO_4	0.75	02	50	Na_3PO_4	0.75	0.00265	**
**02**	25	Na_3PO_4	0.75	02	50	Na_3PO_4	0.75	0.00476	**
**02**	DC	Na_3PO_4	0.075	02	0	Na_3PO_4	0.075	0.02837	*
**02**	DC	Na_3PO_4	0.075	02	1	Na_3PO_4	0.075	0.00289	**
**02**	DC	Na_3PO_4	0.075	02	5	Na_3PO_4	0.075	0.00090	***
**02**	DC	Na_3PO_4	0.075	02	10	Na_3PO_4	0.075	0.00257	**
**02**	DC	Na_3PO_4	0.075	02	25	Na_3PO_4	0.075	0.00000	***
**02**	DC	Na_3PO_4	0.075	02	50	Na_3PO_4	0.075	0.00000	***
**02**	0	Na_3PO_4	0.075	02	1	Na_3PO_4	0.075	0.00464	**
**02**	0	Na_3PO_4	0.075	02	5	Na_3PO_4	0.075	0.00008	***
**02**	0	Na_3PO_4	0.075	02	10	Na_3PO_4	0.075	0.00197	**
**02**	0	Na_3PO_4	0.075	02	25	Na_3PO_4	0.075	0.00005	***
**02**	0	Na_3PO_4	0.075	02	50	Na_3PO_4	0.075	0.00005	***
**02**	1	Na_3PO_4	0.075	02	5	Na_3PO_4	0.075	0.00024	***
**02**	1	Na_3PO_4	0.075	02	10	Na_3PO_4	0.075	0.00270	**
**02**	1	Na_3PO_4	0.075	02	25	Na_3PO_4	0.075	0.00004	***
**02**	1	Na_3PO_4	0.075	02	50	Na_3PO_4	0.075	0.00004	***
**02**	5	Na_3PO_4	0.075	02	10	Na_3PO_4	0.075	0.00730	**
**02**	5	Na_3PO_4	0.075	02	25	Na_3PO_4	0.075	0.00021	***
**02**	5	Na_3PO_4	0.075	02	50	Na_3PO_4	0.075	0.00021	***
**02**	10	Na_3PO_4	0.075	02	25	Na_3PO_4	0.075	0.00379	**
**02**	10	Na_3PO_4	0.075	02	50	Na_3PO_4	0.075	0.00379	**
**02**	25	Na_3PO_4	0.075	02	50	Na_3PO_4	0.075	Nd	Nd
**06**	DC	Na_2CO_3	75	06	DC	Na_3PO_4	75	0.00015	***
**06**	0	Na_2CO_3	75	06	0	Na_3PO_4	75	0.02838	*

**06**	1	Na_2CO_3	75	06	1	Na_3PO_4	75	0.02324	*
**06**	5	Na_2CO_3	75	06	5	Na_3PO_4	75	0.24258	Ns
**06**	10	Na_2CO_3	75	06	10	Na_3PO_4	75	0.90797	Ns
**06**	25	Na_2CO_3	75	06	25	Na_3PO_4	75	0.01324	*
**06**	50	Na_2CO_3	75	06	50	Na_3PO_4	75	0.15942	Ns
**06**	DC	Na_2CO_3	7.5	06	DC	Na_3PO_4	7.5	0.73907	Ns
**06**	0	Na_2CO_3	7.5	06	0	Na_3PO_4	7.5	0.77525	Ns
**06**	1	Na_2CO_3	7.5	06	1	Na_3PO_4	7.5	0.35535	Ns
**06**	5	Na_2CO_3	7.5	06	5	Na_3PO_4	7.5	0.09819	Ns
**06**	10	Na_2CO_3	7.5	06	10	Na_3PO_4	7.5	0.12957	Ns
**06**	25	Na_2CO_3	7.5	06	25	Na_3PO_4	7.5	0.05566	Ns
**06**	50	Na_2CO_3	7.5	06	50	Na_3PO_4	7.5	0.11565	Ns
**06**	DC	Na_2CO_3	0.75	06	DC	Na_3PO_4	0.75	0.06662	Ns
**06**	0	Na_2CO_3	0.75	06	0	Na_3PO_4	0.75	0.00903	**
**06**	1	Na_2CO_3	0.75	06	1	Na_3PO_4	0.75	0.01917	*
**06**	5	Na_2CO_3	0.75	06	5	Na_3PO_4	0.75	0.00055	***
**06**	10	Na_2CO_3	0.75	06	10	Na_3PO_4	0.75	0.00001	***
**06**	25	Na_2CO_3	0.75	06	25	Na_3PO_4	0.75	0.96521	Ns
**06**	50	Na_2CO_3	0.75	06	50	Na_3PO_4	0.75	0.18647	Ns
**06**	DC	Na_2CO_3	0.075	06	DC	Na_3PO_4	0.075	0.04916	*
**06**	0	Na_2CO_3	0.075	06	0	Na_3PO_4	0.075	0.00522	**
**06**	1	Na_2CO_3	0.075	06	1	Na_3PO_4	0.075	0.00257	**
**06**	5	Na_2CO_3	0.075	06	5	Na_3PO_4	0.075	0.00203	**
**06**	10	Na_2CO_3	0.075	06	10	Na_3PO_4	0.075	0.00305	**
**06**	25	Na_2CO_3	0.075	06	25	Na_3PO_4	0.075	0.00284	**
**06**	50	Na_2CO_3	0.075	06	50	Na_3PO_4	0.075	0.00400	**
**02**	DC	Na_2CO_3	75	02	DC	Na_3PO_4	75	0.84499	Ns
**02**	0	Na_2CO_3	75	02	0	Na_3PO_4	75	0.09852	Ns
**02**	1	Na_2CO_3	75	02	1	Na_3PO_4	75	0.24368	Ns
**02**	5	Na_2CO_3	75	02	5	Na_3PO_4	75	0.10991	Ns
**02**	10	Na_2CO_3	75	02	10	Na_3PO_4	75	0.08386	Ns
**02**	25	Na_2CO_3	75	02	25	Na_3PO_4	75	0.12868	Ns
**02**	50	Na_2CO_3	75	02	50	Na_3PO_4	75	0.01204	*
**02**	DC	Na_2CO_3	7.5	02	DC	Na_3PO_4	7.5	0.74152	Ns
**02**	0	Na_2CO_3	7.5	02	0	Na_3PO_4	7.5	0.09797	Ns
**02**	1	Na_2CO_3	7.5	02	1	Na_3PO_4	7.5	0.91070	Ns
**02**	5	Na_2CO_3	7.5	02	5	Na_3PO_4	7.5	0.26768	Ns
**02**	10	Na_2CO_3	7.5	02	10	Na_3PO_4	7.5	0.81429	Ns
**02**	25	Na_2CO_3	7.5	02	25	Na_3PO_4	7.5	0.06639	Ns
**02**	50	Na_2CO_3	7.5	02	50	Na_3PO_4	7.5	0.52196	Ns
**02**	DC	Na_2CO_3	0.75	02	DC	Na_3PO_4	0.75	0.04131	*
**02**	0	Na_2CO_3	0.75	02	0	Na_3PO_4	0.75	0.00364	**
**02**	1	Na_2CO_3	0.75	02	1	Na_3PO_4	0.75	0.00846	**
**02**	5	Na_2CO_3	0.75	02	5	Na_3PO_4	0.75	0.00145	**
**02**	10	Na_2CO_3	0.75	02	10	Na_3PO_4	0.75	0.00327	**
**02**	25	Na_2CO_3	0.75	02	25	Na_3PO_4	0.75	0.00209	**
**02**	50	Na_2CO_3	0.75	02	50	Na_3PO_4	0.75	0.00300	**
**02**	DC	Na_2CO_3	0.075	02	DC	Na_3PO_4	0.075	0.27247	Ns
**02**	0	Na_2CO_3	0.075	02	0	Na_3PO_4	0.075	0.19906	Ns
**02**	1	Na_2CO_3	0.075	02	1	Na_3PO_4	0.075	0.65944	Ns
**02**	5	Na_2CO_3	0.075	02	5	Na_3PO_4	0.075	0.00024	***

**02**	10	Na_2CO_3	0.075	02	10	Na_3PO_4	0.075	0.01143	*
**02**	25	Na_2CO_3	0.075	02	25	Na_3PO_4	0.075	0.00997	**
**02**	50	Na_2CO_3	0.075	02	50	Na_3PO_4	0.075	Nd	Nd
**06**	DC	Na_2CO_3	75	02	DC	Na_2CO_3	75	0.02191	*
**06**	0	Na_2CO_3	75	02	0	Na_2CO_3	75	0.01296	*
**06**	1	Na_2CO_3	75	02	1	Na_2CO_3	75	0.00926	**
**06**	5	Na_2CO_3	75	02	5	Na_2CO_3	75	0.00866	**
**06**	10	Na_2CO_3	75	02	10	Na_2CO_3	75	0.01008	*
**06**	25	Na_2CO_3	75	02	25	Na_2CO_3	75	0.00467	**
**06**	50	Na_2CO_3	75	02	50	Na_2CO_3	75	0.02418	*
**06**	DC	Na_2CO_3	7.5	02	DC	Na_2CO_3	7.5	0.37563	Ns
**06**	0	Na_2CO_3	7.5	02	0	Na_2CO_3	7.5	0.00277	**
**06**	1	Na_2CO_3	7.5	02	1	Na_2CO_3	7.5	0.00165	**
**06**	5	Na_2CO_3	7.5	02	5	Na_2CO_3	7.5	0.00146	**
**06**	10	Na_2CO_3	7.5	02	10	Na_2CO_3	7.5	0.00221	**
**06**	25	Na_2CO_3	7.5	02	25	Na_2CO_3	7.5	0.00069	***
**06**	50	Na_2CO_3	7.5	02	50	Na_2CO_3	7.5	0.00153	**
**06**	DC	Na_2CO_3	0.75	02	DC	Na_2CO_3	0.75	0.03282	*
**06**	0	Na_2CO_3	0.75	02	0	Na_2CO_3	0.75	0.00103	**
**06**	1	Na_2CO_3	0.75	02	1	Na_2CO_3	0.75	0.00008	***
**06**	5	Na_2CO_3	0.75	02	5	Na_2CO_3	0.75	0.00191	**
**06**	10	Na_2CO_3	0.75	02	10	Na_2CO_3	0.75	0.00062	***
**06**	25	Na_2CO_3	0.75	02	25	Na_2CO_3	0.75	0.03525	*
**06**	50	Na_2CO_3	0.75	02	50	Na_2CO_3	0.75	0.72325	Ns
**06**	DC	Na_2CO_3	0.075	02	DC	Na_2CO_3	0.075	0.01474	*
**06**	0	Na_2CO_3	0.075	02	0	Na_2CO_3	0.075	0.03352	*
**06**	1	Na_2CO_3	0.075	02	1	Na_2CO_3	0.075	0.00097	***
**06**	5	Na_2CO_3	0.075	02	5	Na_2CO_3	0.075	0.00060	***
**06**	10	Na_2CO_3	0.075	02	10	Na_2CO_3	0.075	0.00224	**
**06**	25	Na_2CO_3	0.075	02	25	Na_2CO_3	0.075	0.66111	Ns
**06**	50	Na_2CO_3	0.075	02	50	Na_2CO_3	0.075	0.00400	**
**06**	DC	Na_3PO_4	75	02	DC	Na_3PO_4	75	0.75785	Ns
**06**	0	Na_3PO_4	75	02	0	Na_3PO_4	75	0.12568	Ns
**06**	1	Na_3PO_4	75	02	1	Na_3PO_4	75	0.33299	Ns
**06**	5	Na_3PO_4	75	02	5	Na_3PO_4	75	0.21983	Ns
**06**	10	Na_3PO_4	75	02	10	Na_3PO_4	75	0.28763	Ns
**06**	25	Na_3PO_4	75	02	25	Na_3PO_4	75	0.43078	Ns
**06**	50	Na_3PO_4	75	02	50	Na_3PO_4	75	0.38240	Ns
**06**	DC	Na_3PO_4	7.5	02	DC	Na_3PO_4	7.5	0.15451	Ns
**06**	0	Na_3PO_4	7.5	02	0	Na_3PO_4	7.5	0.00510	**
**06**	1	Na_3PO_4	7.5	02	1	Na_3PO_4	7.5	0.00052	***
**06**	5	Na_3PO_4	7.5	02	5	Na_3PO_4	7.5	0.00115	**
**06**	10	Na_3PO_4	7.5	02	10	Na_3PO_4	7.5	0.00251	**
**06**	25	Na_3PO_4	7.5	02	25	Na_3PO_4	7.5	0.00005	***
**06**	50	Na_3PO_4	7.5	02	50	Na_3PO_4	7.5	0.00007	***
**06**	DC	Na_3PO_4	0.75	02	DC	Na_3PO_4	0.75	0.01269	*
**06**	0	Na_3PO_4	0.75	02	0	Na_3PO_4	0.75	0.00166	**
**06**	1	Na_3PO_4	0.75	02	1	Na_3PO_4	0.75	0.00242	**
**06**	5	Na_3PO_4	0.75	02	5	Na_3PO_4	0.75	0.06008	Ns
**06**	10	Na_3PO_4	0.75	02	10	Na_3PO_4	0.75	0.03670	*
**06**	25	Na_3PO_4	0.75	02	25	Na_3PO_4	0.75	0.05312	Ns

**06**	50	Na_3PO_4	0.75	02	50	Na_3PO_4	0.75	0.00039	***
**06**	DC	Na_3PO_4	0.075	02	DC	Na_3PO_4	0.075	0.72915	Ns
**06**	0	Na_3PO_4	0.075	02	0	Na_3PO_4	0.075	0.17862	Ns
**06**	1	Na_3PO_4	0.075	02	1	Na_3PO_4	0.075	0.00560	**
**06**	5	Na_3PO_4	0.075	02	5	Na_3PO_4	0.075	0.00083	***
**06**	10	Na_3PO_4	0.075	02	10	Na_3PO_4	0.075	0.01701	*
**06**	25	Na_3PO_4	0.075	02	25	Na_3PO_4	0.075	Nd	Nd
**06**	50	Na_3PO_4	0.075	02	50	Na_3PO_4	0.075	Nd	Nd
