# Supplementary material for: Interplay of phosphate and carbonate ions with flavin photosensitizers in photodynamic inactivation of bacteria
Source: PLoS One. 2021 Jun 11;16(6):e0253212. doi: 10.1371/journal.pone.0253212 (PMC8195418; doi:10.1371/journal.pone.0253212)
Supplement: S4 File — (PDF) [file pone.0253212.s011.pdf]

Statistical analysis of *Pseudomonas aeruginosa* inactivation data

Method

In order to investigate the gathered data statistically, significance between samples was calculated via unpaired, two-tailed t-tests assuming normal distribution. Events were considered statistically significant for $p < 0.05$ and marked in the following table with one asterisk. When p is < 0.01, events were considered highly significant and marked with two asterisks. Extremely significant events with $p < 0.001$ were marked with three asterisks. Non-significant events were marked with “Ns”, whenever a calculation of the corresponding p-value was not possible the values were marked with “Nd”.

Results

Table 1: Results of the performed t-tests for each of the listed conditions. The abbreviations represent the following: DC indicates the dark control, numbers in the same column represent the applied PS concentration in µmol l^{-1}. 06 indicates the PS FLASH-06a while 02 indicates FLASH-02a. Applied salts are abbreviated as empirical formulas with their respective concentration in the next column.

**Condition 1**				**Vs.**	**Condition 2**				**p**	**significance**
**06**	DC	Na_2CO_3	75		06	0	Na_2CO_3	75	0.02515	*
**06**	DC	Na_2CO_3	75		06	1	Na_2CO_3	75	0.00112	**
**06**	DC	Na_2CO_3	75		06	5	Na_2CO_3	75	0.00011	***
**06**	DC	Na_2CO_3	75		06	10	Na_2CO_3	75	0.00048	***
**06**	DC	Na_2CO_3	75		06	25	Na_2CO_3	75	0.00028	***
**06**	DC	Na_2CO_3	75		06	50	Na_2CO_3	75	0.00009	***
**06**	0	Na_2CO_3	75		06	1	Na_2CO_3	75	0.39465	Ns
**06**	0	Na_2CO_3	75		06	5	Na_2CO_3	75	0.95794	Ns
**06**	0	Na_2CO_3	75		06	10	Na_2CO_3	75	0.35025	Ns
**06**	0	Na_2CO_3	75		06	25	Na_2CO_3	75	0.75719	Ns
**06**	0	Na_2CO_3	75		06	50	Na_2CO_3	75	0.68071	Ns
**06**	1	Na_2CO_3	75		06	5	Na_2CO_3	75	0.09524	Ns
**06**	1	Na_2CO_3	75		06	10	Na_2CO_3	75	0.81546	Ns
**06**	1	Na_2CO_3	75		06	25	Na_2CO_3	75	0.21598	Ns
**06**	1	Na_2CO_3	75		06	50	Na_2CO_3	75	0.03209	*
**06**	5	Na_2CO_3	75		06	10	Na_2CO_3	75	0.04376	*
**06**	5	Na_2CO_3	75		06	25	Na_2CO_3	75	0.49085	Ns
**06**	5	Na_2CO_3	75		06	50	Na_2CO_3	75	0.20335	Ns
**06**	10	Na_2CO_3	75		06	25	Na_2CO_3	75	0.12428	Ns
**06**	10	Na_2CO_3	75		06	50	Na_2CO_3	75	0.01400	*
**06**	25	Na_2CO_3	75		06	50	Na_2CO_3	75	0.11005	Ns
**06**	DC	Na_2CO_3	7.5		06	0	Na_2CO_3	7.5	0.45757	Ns
**06**	DC	Na_2CO_3	7.5		06	1	Na_2CO_3	7.5	0.04401	*
**06**	DC	Na_2CO_3	7.5		06	5	Na_2CO_3	7.5	0.03305	*
**06**	DC	Na_2CO_3	7.5		06	10	Na_2CO_3	7.5	0.00088	***
**06**	DC	Na_2CO_3	7.5		06	25	Na_2CO_3	7.5	0.00014	***
**06**	DC	Na_2CO_3	7.5		06	50	Na_2CO_3	7.5	0.00000	***
**06**	0	Na_2CO_3	7.5		06	1	Na_2CO_3	7.5	0.10114	Ns
**06**	0	Na_2CO_3	7.5		06	5	Na_2CO_3	7.5	0.06555	Ns
**06**	0	Na_2CO_3	7.5		06	10	Na_2CO_3	7.5	0.00088	***
**06**	0	Na_2CO_3	7.5		06	25	Na_2CO_3	7.5	0.00011	***
**06**	0	Na_2CO_3	7.5		06	50	Na_2CO_3	7.5	0.00000	***
**06**	1	Na_2CO_3	7.5		06	5	Na_2CO_3	7.5	0.71994	Ns
**06**	1	Na_2CO_3	7.5		06	10	Na_2CO_3	7.5	0.00073	***

**06**	1	Na_2CO_3	7.5	06	25	Na_2CO_3	7.5	0.00005	***
**06**	1	Na_2CO_3	7.5	06	50	Na_2CO_3	7.5	0.00000	***
**06**	5	Na_2CO_3	7.5	06	10	Na_2CO_3	7.5	0.00027	***
**06**	5	Na_2CO_3	7.5	06	25	Na_2CO_3	7.5	0.00000	***
**06**	5	Na_2CO_3	7.5	06	50	Na_2CO_3	7.5	0.00000	***
**06**	10	Na_2CO_3	7.5	06	25	Na_2CO_3	7.5	0.00001	***
**06**	10	Na_2CO_3	7.5	06	50	Na_2CO_3	7.5	0.00000	***
**06**	25	Na_2CO_3	7.5	06	50	Na_2CO_3	7.5	0.00001	***
**06**	DC	Na_2CO_3	0.75	06	0	Na_2CO_3	0.75	0.37773	Ns
**06**	DC	Na_2CO_3	0.75	06	1	Na_2CO_3	0.75	0.21927	Ns
**06**	DC	Na_2CO_3	0.75	06	5	Na_2CO_3	0.75	0.00314	**
**06**	DC	Na_2CO_3	0.75	06	10	Na_2CO_3	0.75	0.00478	**
**06**	DC	Na_2CO_3	0.75	06	25	Na_2CO_3	0.75	0.00212	**
**06**	DC	Na_2CO_3	0.75	06	50	Na_2CO_3	0.75	0.00393	**
**06**	0	Na_2CO_3	0.75	06	1	Na_2CO_3	0.75	0.15875	Ns
**06**	0	Na_2CO_3	0.75	06	5	Na_2CO_3	0.75	0.00061	***
**06**	0	Na_2CO_3	0.75	06	10	Na_2CO_3	0.75	0.00036	***
**06**	0	Na_2CO_3	0.75	06	25	Na_2CO_3	0.75	0.00001	***
**06**	0	Na_2CO_3	0.75	06	50	Na_2CO_3	0.75	0.00031	***
**06**	1	Na_2CO_3	0.75	06	5	Na_2CO_3	0.75	0.00094	***
**06**	1	Na_2CO_3	0.75	06	10	Na_2CO_3	0.75	0.00058	***
**06**	1	Na_2CO_3	0.75	06	25	Na_2CO_3	0.75	0.00001	***
**06**	1	Na_2CO_3	0.75	06	50	Na_2CO_3	0.75	0.00029	***
**06**	5	Na_2CO_3	0.75	06	10	Na_2CO_3	0.75	0.35203	Ns
**06**	5	Na_2CO_3	0.75	06	25	Na_2CO_3	0.75	0.00176	**
**06**	5	Na_2CO_3	0.75	06	50	Na_2CO_3	0.75	0.00722	**
**06**	10	Na_2CO_3	0.75	06	25	Na_2CO_3	0.75	0.00057	***
**06**	10	Na_2CO_3	0.75	06	50	Na_2CO_3	0.75	0.00437	**
**06**	25	Na_2CO_3	0.75	06	50	Na_2CO_3	0.75	0.31449	Ns
**06**	DC	Na_2CO_3	0.075	06	0	Na_2CO_3	0.075	0.35378	Ns
**06**	DC	Na_2CO_3	0.075	06	1	Na_2CO_3	0.075	0.01921	*
**06**	DC	Na_2CO_3	0.075	06	5	Na_2CO_3	0.075	0.00116	**
**06**	DC	Na_2CO_3	0.075	06	10	Na_2CO_3	0.075	0.00077	***
**06**	DC	Na_2CO_3	0.075	06	25	Na_2CO_3	0.075	0.00009	***
**06**	DC	Na_2CO_3	0.075	06	50	Na_2CO_3	0.075	0.00009	***
**06**	0	Na_2CO_3	0.075	06	1	Na_2CO_3	0.075	0.02918	*
**06**	0	Na_2CO_3	0.075	06	5	Na_2CO_3	0.075	0.00132	**
**06**	0	Na_2CO_3	0.075	06	10	Na_2CO_3	0.075	0.00089	***
**06**	0	Na_2CO_3	0.075	06	25	Na_2CO_3	0.075	0.00008	***
**06**	0	Na_2CO_3	0.075	06	50	Na_2CO_3	0.075	0.00008	***
**06**	1	Na_2CO_3	0.075	06	5	Na_2CO_3	0.075	0.00287	**
**06**	1	Na_2CO_3	0.075	06	10	Na_2CO_3	0.075	0.00212	**
**06**	1	Na_2CO_3	0.075	06	25	Na_2CO_3	0.075	0.00001	***
**06**	1	Na_2CO_3	0.075	06	50	Na_2CO_3	0.075	0.00001	***
**06**	5	Na_2CO_3	0.075	06	10	Na_2CO_3	0.075	0.25114	Ns
**06**	5	Na_2CO_3	0.075	06	25	Na_2CO_3	0.075	0.00613	**
**06**	5	Na_2CO_3	0.075	06	50	Na_2CO_3	0.075	0.00613	**
**06**	10	Na_2CO_3	0.075	06	25	Na_2CO_3	0.075	0.00758	**
**06**	10	Na_2CO_3	0.075	06	50	Na_2CO_3	0.075	0.00758	**
**06**	25	Na_2CO_3	0.075	06	50	Na_2CO_3	0.075	Nd	Nd
**06**	DC	Na_3PO_4	75	06	0	Na_3PO_4	75	0.07426	Ns

**06**	DC	Na_3PO_4	75	06	1	Na_3PO_4	75	0.06734	Ns
**06**	DC	Na_3PO_4	75	06	5	Na_3PO_4	75	0.03055	*
**06**	DC	Na_3PO_4	75	06	10	Na_3PO_4	75	0.00797	**
**06**	DC	Na_3PO_4	75	06	25	Na_3PO_4	75	0.00536	**
**06**	DC	Na_3PO_4	75	06	50	Na_3PO_4	75	0.00177	**
**06**	0	Na_3PO_4	75	06	1	Na_3PO_4	75	0.80887	Ns
**06**	0	Na_3PO_4	75	06	5	Na_3PO_4	75	0.64656	Ns
**06**	0	Na_3PO_4	75	06	10	Na_3PO_4	75	0.10294	Ns
**06**	0	Na_3PO_4	75	06	25	Na_3PO_4	75	0.05167	Ns
**06**	0	Na_3PO_4	75	06	50	Na_3PO_4	75	0.00901	**
**06**	1	Na_3PO_4	75	06	5	Na_3PO_4	75	0.39657	Ns
**06**	1	Na_3PO_4	75	06	10	Na_3PO_4	75	0.04034	*
**06**	1	Na_3PO_4	75	06	25	Na_3PO_4	75	0.01611	*
**06**	1	Na_3PO_4	75	06	50	Na_3PO_4	75	0.00189	**
**06**	5	Na_3PO_4	75	06	10	Na_3PO_4	75	0.09765	Ns
**06**	5	Na_3PO_4	75	06	25	Na_3PO_4	75	0.03235	*
**06**	5	Na_3PO_4	75	06	50	Na_3PO_4	75	0.00229	**
**06**	10	Na_3PO_4	75	06	25	Na_3PO_4	75	0.41766	Ns
**06**	10	Na_3PO_4	75	06	50	Na_3PO_4	75	0.01168	*
**06**	25	Na_3PO_4	75	06	50	Na_3PO_4	75	0.01827	*
**06**	DC	Na_3PO_4	7.5	06	0	Na_3PO_4	7.5	0.92422	Ns
**06**	DC	Na_3PO_4	7.5	06	1	Na_3PO_4	7.5	0.80217	*
**06**	DC	Na_3PO_4	7.5	06	5	Na_3PO_4	7.5	0.00030	***
**06**	DC	Na_3PO_4	7.5	06	10	Na_3PO_4	7.5	0.00013	***
**06**	DC	Na_3PO_4	7.5	06	25	Na_3PO_4	7.5	0.00002	***
**06**	DC	Na_3PO_4	7.5	06	50	Na_3PO_4	7.5	0.00002	***
**06**	0	Na_3PO_4	7.5	06	1	Na_3PO_4	7.5	0.79221	Ns
**06**	0	Na_3PO_4	7.5	06	5	Na_3PO_4	7.5	0.00269	**
**06**	0	Na_3PO_4	7.5	06	10	Na_3PO_4	7.5	0.00094	***
**06**	0	Na_3PO_4	7.5	06	25	Na_3PO_4	7.5	0.00052	***
**06**	0	Na_3PO_4	7.5	06	50	Na_3PO_4	7.5	0.00044	***
**06**	1	Na_3PO_4	7.5	06	5	Na_3PO_4	7.5	0.00108	**
**06**	1	Na_3PO_4	7.5	06	10	Na_3PO_4	7.5	0.00040	***
**06**	1	Na_3PO_4	7.5	06	25	Na_3PO_4	7.5	0.00013	***
**06**	1	Na_3PO_4	7.5	06	50	Na_3PO_4	7.5	0.00013	***
**06**	5	Na_3PO_4	7.5	06	10	Na_3PO_4	7.5	0.00000	***
**06**	5	Na_3PO_4	7.5	06	25	Na_3PO_4	7.5	0.00000	***
**06**	5	Na_3PO_4	7.5	06	50	Na_3PO_4	7.5	0.00000	***
**06**	10	Na_3PO_4	7.5	06	25	Na_3PO_4	7.5	0.00142	**
**06**	10	Na_3PO_4	7.5	06	50	Na_3PO_4	7.5	0.00003	***
**06**	25	Na_3PO_4	7.5	06	50	Na_3PO_4	7.5	0.00010	***
**06**	DC	Na_3PO_4	0.75	06	0	Na_3PO_4	0.75	0.08926	Ns
**06**	DC	Na_3PO_4	0.75	06	1	Na_3PO_4	0.75	0.05896	Ns
**06**	DC	Na_3PO_4	0.75	06	5	Na_3PO_4	0.75	0.00004	***
**06**	DC	Na_3PO_4	0.75	06	10	Na_3PO_4	0.75	0.00001	***
**06**	DC	Na_3PO_4	0.75	06	25	Na_3PO_4	0.75	0.00016	***
**06**	DC	Na_3PO_4	0.75	06	50	Na_3PO_4	0.75	0.00016	***
**06**	0	Na_3PO_4	0.75	06	1	Na_3PO_4	0.75	0.55883	Ns
**06**	0	Na_3PO_4	0.75	06	5	Na_3PO_4	0.75	0.00002	***
**06**	0	Na_3PO_4	0.75	06	10	Na_3PO_4	0.75	0.00003	***
**06**	0	Na_3PO_4	0.75	06	25	Na_3PO_4	0.75	0.00009	***

**06**	0	Na_3PO_4	0.75	06	50	Na_3PO_4	0.75	0.00009	***
**06**	1	Na_3PO_4	0.75	06	5	Na_3PO_4	0.75	0.00006	***
**06**	1	Na_3PO_4	0.75	06	10	Na_3PO_4	0.75	0.00023	***
**06**	1	Na_3PO_4	0.75	06	25	Na_3PO_4	0.75	0.00003	***
**06**	1	Na_3PO_4	0.75	06	50	Na_3PO_4	0.75	0.00003	***
**06**	5	Na_3PO_4	0.75	06	10	Na_3PO_4	0.75	0.00282	**
**06**	5	Na_3PO_4	0.75	06	25	Na_3PO_4	0.75	0.00022	***
**06**	5	Na_3PO_4	0.75	06	50	Na_3PO_4	0.75	0.00022	***
**06**	10	Na_3PO_4	0.75	06	25	Na_3PO_4	0.75	0.00071	***
**06**	10	Na_3PO_4	0.75	06	50	Na_3PO_4	0.75	0.00071	***
**06**	25	Na_3PO_4	0.75	06	50	Na_3PO_4	0.75	Nd	Nd
**06**	DC	Na_3PO_4	0.075	06	0	Na_3PO_4	0.075	0.06977	Ns
**06**	DC	Na_3PO_4	0.075	06	1	Na_3PO_4	0.075	0.00481	**
**06**	DC	Na_3PO_4	0.075	06	5	Na_3PO_4	0.075	0.00698	**
**06**	DC	Na_3PO_4	0.075	06	10	Na_3PO_4	0.075	0.00000	***
**06**	DC	Na_3PO_4	0.075	06	25	Na_3PO_4	0.075	0.00000	***
**06**	DC	Na_3PO_4	0.075	06	50	Na_3PO_4	0.075	0.00000	***
**06**	0	Na_3PO_4	0.075	06	1	Na_3PO_4	0.075	0.05887	Ns
**06**	0	Na_3PO_4	0.075	06	5	Na_3PO_4	0.075	0.00694	**
**06**	0	Na_3PO_4	0.075	06	10	Na_3PO_4	0.075	0.00003	***
**06**	0	Na_3PO_4	0.075	06	25	Na_3PO_4	0.075	0.00003	***
**06**	0	Na_3PO_4	0.075	06	50	Na_3PO_4	0.075	0.00003	***
**06**	1	Na_3PO_4	0.075	06	5	Na_3PO_4	0.075	0.00850	**
**06**	1	Na_3PO_4	0.075	06	10	Na_3PO_4	0.075	0.00002	***
**06**	1	Na_3PO_4	0.075	06	25	Na_3PO_4	0.075	0.00002	***
**06**	1	Na_3PO_4	0.075	06	50	Na_3PO_4	0.075	0.00002	***
**06**	5	Na_3PO_4	0.075	06	10	Na_3PO_4	0.075	0.00528	**
**06**	5	Na_3PO_4	0.075	06	25	Na_3PO_4	0.075	0.00528	**
**06**	5	Na_3PO_4	0.075	06	50	Na_3PO_4	0.075	0.00528	**
**06**	10	Na_3PO_4	0.075	06	25	Na_3PO_4	0.075	1.00000	Ns
**06**	10	Na_3PO_4	0.075	06	50	Na_3PO_4	0.075	1.00000	Ns
**06**	25	Na_3PO_4	0.075	06	50	Na_3PO_4	0.075	1.00000	Ns
**02**	DC	Na_2CO_3	75	02	0	Na_2CO_3	75	0.00243	**
**02**	DC	Na_2CO_3	75	02	1	Na_2CO_3	75	0.00204	**
**02**	DC	Na_2CO_3	75	02	5	Na_2CO_3	75	0.00307	**
**02**	DC	Na_2CO_3	75	02	10	Na_2CO_3	75	0.00204	**
**02**	DC	Na_2CO_3	75	02	25	Na_2CO_3	75	0.00320	**
**02**	DC	Na_2CO_3	75	02	50	Na_2CO_3	75	0.00312	**
**02**	0	Na_2CO_3	75	02	1	Na_2CO_3	75	0.37289	Ns
**02**	0	Na_2CO_3	75	02	5	Na_2CO_3	75	0.33219	Ns
**02**	0	Na_2CO_3	75	02	10	Na_2CO_3	75	0.37289	Ns
**02**	0	Na_2CO_3	75	02	25	Na_2CO_3	75	0.06053	Ns
**02**	0	Na_2CO_3	75	02	50	Na_2CO_3	75	0.08106	Ns
**02**	1	Na_2CO_3	75	02	5	Na_2CO_3	75	0.83054	Ns
**02**	1	Na_2CO_3	75	02	10	Na_2CO_3	75	1.00000	Ns
**02**	1	Na_2CO_3	75	02	25	Na_2CO_3	75	0.01234	*
**02**	1	Na_2CO_3	75	02	50	Na_2CO_3	75	0.02059	*
**02**	5	Na_2CO_3	75	02	10	Na_2CO_3	75	0.83054	Ns
**02**	5	Na_2CO_3	75	02	25	Na_2CO_3	75	0.00487	**
**02**	5	Na_2CO_3	75	02	50	Na_2CO_3	75	0.00939	**
**02**	10	Na_2CO_3	75	02	25	Na_2CO_3	75	0.01234	*

**02**	10	Na_2CO_3	75	02	50	Na_2CO_3	75	0.02059	*
**02**	25	Na_2CO_3	75	02	50	Na_2CO_3	75	0.10226	Ns
**02**	DC	Na_2CO_3	7.5	02	0	Na_2CO_3	7.5	0.00836	**
**02**	DC	Na_2CO_3	7.5	02	1	Na_2CO_3	7.5	0.00006	***
**02**	DC	Na_2CO_3	7.5	02	5	Na_2CO_3	7.5	0.00034	***
**02**	DC	Na_2CO_3	7.5	02	10	Na_2CO_3	7.5	0.00098	***
**02**	DC	Na_2CO_3	7.5	02	25	Na_2CO_3	7.5	0.03810	*
**02**	DC	Na_2CO_3	7.5	02	50	Na_2CO_3	7.5	0.00136	**
**02**	0	Na_2CO_3	7.5	02	1	Na_2CO_3	7.5	0.01355	*
**02**	0	Na_2CO_3	7.5	02	5	Na_2CO_3	7.5	0.00012	***
**02**	0	Na_2CO_3	7.5	02	10	Na_2CO_3	7.5	0.00045	***
**02**	0	Na_2CO_3	7.5	02	25	Na_2CO_3	7.5	0.05053	Ns
**02**	0	Na_2CO_3	7.5	02	50	Na_2CO_3	7.5	0.00080	***
**02**	1	Na_2CO_3	7.5	02	5	Na_2CO_3	7.5	0.00148	**
**02**	1	Na_2CO_3	7.5	02	10	Na_2CO_3	7.5	0.00197	**
**02**	1	Na_2CO_3	7.5	02	25	Na_2CO_3	7.5	0.06990	Ns
**02**	1	Na_2CO_3	7.5	02	50	Na_2CO_3	7.5	0.00252	**
**02**	5	Na_2CO_3	7.5	02	10	Na_2CO_3	7.5	0.00313	**
**02**	5	Na_2CO_3	7.5	02	25	Na_2CO_3	7.5	0.30435	Ns
**02**	5	Na_2CO_3	7.5	02	50	Na_2CO_3	7.5	0.00397	**
**02**	10	Na_2CO_3	7.5	02	25	Na_2CO_3	7.5	0.51353	Ns
**02**	10	Na_2CO_3	7.5	02	50	Na_2CO_3	7.5	0.41174	Ns
**02**	25	Na_2CO_3	7.5	02	50	Na_2CO_3	7.5	0.37981	Ns
**02**	DC	Na_2CO_3	0.75	02	0	Na_2CO_3	0.75	0.01790	*
**02**	DC	Na_2CO_3	0.75	02	1	Na_2CO_3	0.75	0.00003	***
**02**	DC	Na_2CO_3	0.75	02	5	Na_2CO_3	0.75	0.00185	**
**02**	DC	Na_2CO_3	0.75	02	10	Na_2CO_3	0.75	0.00117	**
**02**	DC	Na_2CO_3	0.75	02	25	Na_2CO_3	0.75	0.00142	**
**02**	DC	Na_2CO_3	0.75	02	50	Na_2CO_3	0.75	0.00003	***
**02**	0	Na_2CO_3	0.75	02	1	Na_2CO_3	0.75	0.00007	***
**02**	0	Na_2CO_3	0.75	02	5	Na_2CO_3	0.75	0.00166	**
**02**	0	Na_2CO_3	0.75	02	10	Na_2CO_3	0.75	0.00095	***
**02**	0	Na_2CO_3	0.75	02	25	Na_2CO_3	0.75	0.00122	**
**02**	0	Na_2CO_3	0.75	02	50	Na_2CO_3	0.75	0.00007	***
**02**	1	Na_2CO_3	0.75	02	5	Na_2CO_3	0.75	0.00969	**
**02**	1	Na_2CO_3	0.75	02	10	Na_2CO_3	0.75	0.00482	**
**02**	1	Na_2CO_3	0.75	02	25	Na_2CO_3	0.75	0.00455	**
**02**	1	Na_2CO_3	0.75	02	50	Na_2CO_3	0.75	0.00010	***
**02**	5	Na_2CO_3	0.75	02	10	Na_2CO_3	0.75	0.34671	Ns
**02**	5	Na_2CO_3	0.75	02	25	Na_2CO_3	0.75	0.06293	Ns
**02**	5	Na_2CO_3	0.75	02	50	Na_2CO_3	0.75	0.00148	**
**02**	10	Na_2CO_3	0.75	02	25	Na_2CO_3	0.75	0.18213	Ns
**02**	10	Na_2CO_3	0.75	02	50	Na_2CO_3	0.75	0.00140	**
**02**	25	Na_2CO_3	0.75	02	50	Na_2CO_3	0.75	0.00221	**
**02**	DC	Na_2CO_3	0.075	02	0	Na_2CO_3	0.075	0.00665	**
**02**	DC	Na_2CO_3	0.075	02	1	Na_2CO_3	0.075	0.00009	***
**02**	DC	Na_2CO_3	0.075	02	5	Na_2CO_3	0.075	0.00117	**
**02**	DC	Na_2CO_3	0.075	02	10	Na_2CO_3	0.075	0.00087	***
**02**	DC	Na_2CO_3	0.075	02	25	Na_2CO_3	0.075	0.00042	***
**02**	DC	Na_2CO_3	0.075	02	50	Na_2CO_3	0.075	0.00004	***
**02**	0	Na_2CO_3	0.075	02	1	Na_2CO_3	0.075	0.00175	**

**02**	0	Na_2CO_3	0.075	02	5	Na_2CO_3	0.075	0.00096	***
**02**	0	Na_2CO_3	0.075	02	10	Na_2CO_3	0.075	0.00066	***
**02**	0	Na_2CO_3	0.075	02	25	Na_2CO_3	0.075	0.00026	***
**02**	0	Na_2CO_3	0.075	02	50	Na_2CO_3	0.075	0.00010	***
**02**	1	Na_2CO_3	0.075	02	5	Na_2CO_3	0.075	0.00644	**
**02**	1	Na_2CO_3	0.075	02	10	Na_2CO_3	0.075	0.00332	**
**02**	1	Na_2CO_3	0.075	02	25	Na_2CO_3	0.075	0.00180	**
**02**	1	Na_2CO_3	0.075	02	50	Na_2CO_3	0.075	0.00002	***
**02**	5	Na_2CO_3	0.075	02	10	Na_2CO_3	0.075	0.01762	*
**02**	5	Na_2CO_3	0.075	02	25	Na_2CO_3	0.075	0.00401	**
**02**	5	Na_2CO_3	0.075	02	50	Na_2CO_3	0.075	0.00163	**
**02**	10	Na_2CO_3	0.075	02	25	Na_2CO_3	0.075	0.16374	Ns
**02**	10	Na_2CO_3	0.075	02	50	Na_2CO_3	0.075	0.00322	**
**02**	25	Na_2CO_3	0.075	02	50	Na_2CO_3	0.075	0.00326	**
**02**	DC	Na_3PO_4	75	02	0	Na_3PO_4	75	0.01727	*
**02**	DC	Na_3PO_4	75	02	1	Na_3PO_4	75	0.00976	**
**02**	DC	Na_3PO_4	75	02	5	Na_3PO_4	75	0.00455	**
**02**	DC	Na_3PO_4	75	02	10	Na_3PO_4	75	0.02167	*
**02**	DC	Na_3PO_4	75	02	25	Na_3PO_4	75	0.03570	*
**02**	DC	Na_3PO_4	75	02	50	Na_3PO_4	75	0.03692	*
**02**	0	Na_3PO_4	75	02	1	Na_3PO_4	75	0.64564	Ns
**02**	0	Na_3PO_4	75	02	5	Na_3PO_4	75	0.09630	Ns
**02**	0	Na_3PO_4	75	02	10	Na_3PO_4	75	0.04769	*
**02**	0	Na_3PO_4	75	02	25	Na_3PO_4	75	0.04773	*
**02**	0	Na_3PO_4	75	02	50	Na_3PO_4	75	0.04993	*
**02**	1	Na_3PO_4	75	02	5	Na_3PO_4	75	0.15092	Ns
**02**	1	Na_3PO_4	75	02	10	Na_3PO_4	75	0.05559	Ns
**02**	1	Na_3PO_4	75	02	25	Na_3PO_4	75	0.05006	Ns
**02**	1	Na_3PO_4	75	02	50	Na_3PO_4	75	0.05246	Ns
**02**	5	Na_3PO_4	75	02	10	Na_3PO_4	75	0.09154	Ns
**02**	5	Na_3PO_4	75	02	25	Na_3PO_4	75	0.05799	Ns
**02**	5	Na_3PO_4	75	02	50	Na_3PO_4	75	0.06120	Ns
**02**	10	Na_3PO_4	75	02	25	Na_3PO_4	75	0.09755	Ns
**02**	10	Na_3PO_4	75	02	50	Na_3PO_4	75	0.10761	Ns
**02**	25	Na_3PO_4	75	02	50	Na_3PO_4	75	0.90298	Ns
**02**	DC	Na_3PO_4	7.5	02	0	Na_3PO_4	7.5	0.00317	**
**02**	DC	Na_3PO_4	7.5	02	1	Na_3PO_4	7.5	0.00010	***
**02**	DC	Na_3PO_4	7.5	02	5	Na_3PO_4	7.5	0.00000	***
**02**	DC	Na_3PO_4	7.5	02	10	Na_3PO_4	7.5	0.00001	***
**02**	DC	Na_3PO_4	7.5	02	25	Na_3PO_4	7.5	0.00000	***
**02**	DC	Na_3PO_4	7.5	02	50	Na_3PO_4	7.5	0.00003	***
**02**	0	Na_3PO_4	7.5	02	1	Na_3PO_4	7.5	0.00002	***
**02**	0	Na_3PO_4	7.5	02	5	Na_3PO_4	7.5	0.00003	***
**02**	0	Na_3PO_4	7.5	02	10	Na_3PO_4	7.5	0.00001	***
**02**	0	Na_3PO_4	7.5	02	25	Na_3PO_4	7.5	0.00017	***
**02**	0	Na_3PO_4	7.5	02	50	Na_3PO_4	7.5	0.00002	***
**02**	1	Na_3PO_4	7.5	02	5	Na_3PO_4	7.5	0.16590	Ns
**02**	1	Na_3PO_4	7.5	02	10	Na_3PO_4	7.5	0.12219	Ns
**02**	1	Na_3PO_4	7.5	02	25	Na_3PO_4	7.5	0.11868	Ns
**02**	1	Na_3PO_4	7.5	02	50	Na_3PO_4	7.5	0.00486	**
**02**	5	Na_3PO_4	7.5	02	10	Na_3PO_4	7.5	0.65760	Ns

**02**	5	Na_3PO_4	7.5	02	25	Na_3PO_4	7.5	0.46630	Ns
**02**	5	Na_3PO_4	7.5	02	50	Na_3PO_4	7.5	0.00577	**
**02**	10	Na_3PO_4	7.5	02	25	Na_3PO_4	7.5	0.93451	Ns
**02**	10	Na_3PO_4	7.5	02	50	Na_3PO_4	7.5	0.00730	**
**02**	25	Na_3PO_4	7.5	02	50	Na_3PO_4	7.5	0.01190	*
**02**	DC	Na_3PO_4	0.75	02	0	Na_3PO_4	0.75	0.01827	*
**02**	DC	Na_3PO_4	0.75	02	1	Na_3PO_4	0.75	0.00567	**
**02**	DC	Na_3PO_4	0.75	02	5	Na_3PO_4	0.75	0.00231	**
**02**	DC	Na_3PO_4	0.75	02	10	Na_3PO_4	0.75	0.00279	**
**02**	DC	Na_3PO_4	0.75	02	25	Na_3PO_4	0.75	0.00006	***
**02**	DC	Na_3PO_4	0.75	02	50	Na_3PO_4	0.75	0.00006	***
**02**	0	Na_3PO_4	0.75	02	1	Na_3PO_4	0.75	0.00489	**
**02**	0	Na_3PO_4	0.75	02	5	Na_3PO_4	0.75	0.00123	**
**02**	0	Na_3PO_4	0.75	02	10	Na_3PO_4	0.75	0.00200	**
**02**	0	Na_3PO_4	0.75	02	25	Na_3PO_4	0.75	0.00033	***
**02**	0	Na_3PO_4	0.75	02	50	Na_3PO_4	0.75	0.00033	***
**02**	1	Na_3PO_4	0.75	02	5	Na_3PO_4	0.75	0.01365	*
**02**	1	Na_3PO_4	0.75	02	10	Na_3PO_4	0.75	0.00260	**
**02**	1	Na_3PO_4	0.75	02	25	Na_3PO_4	0.75	0.00378	**
**02**	1	Na_3PO_4	0.75	02	50	Na_3PO_4	0.75	0.00378	**
**02**	5	Na_3PO_4	0.75	02	10	Na_3PO_4	0.75	0.01986	*
**02**	5	Na_3PO_4	0.75	02	25	Na_3PO_4	0.75	0.00940	**
**02**	5	Na_3PO_4	0.75	02	50	Na_3PO_4	0.75	0.00940	**
**02**	10	Na_3PO_4	0.75	02	25	Na_3PO_4	0.75	0.14010	Ns
**02**	10	Na_3PO_4	0.75	02	50	Na_3PO_4	0.75	0.14010	Ns
**02**	25	Na_3PO_4	0.75	02	50	Na_3PO_4	0.75	Nd	Nd
**02**	DC	Na_3PO_4	0.075	02	0	Na_3PO_4	0.075	0.13732	Ns
**02**	DC	Na_3PO_4	0.075	02	1	Na_3PO_4	0.075	0.00072	***
**02**	DC	Na_3PO_4	0.075	02	5	Na_3PO_4	0.075	0.00004	***
**02**	DC	Na_3PO_4	0.075	02	10	Na_3PO_4	0.075	0.00011	***
**02**	DC	Na_3PO_4	0.075	02	25	Na_3PO_4	0.075	0.00011	***
**02**	DC	Na_3PO_4	0.075	02	50	Na_3PO_4	0.075	0.00011	***
**02**	0	Na_3PO_4	0.075	02	1	Na_3PO_4	0.075	0.00272	**
**02**	0	Na_3PO_4	0.075	02	5	Na_3PO_4	0.075	0.00003	***
**02**	0	Na_3PO_4	0.075	02	10	Na_3PO_4	0.075	0.00025	***
**02**	0	Na_3PO_4	0.075	02	25	Na_3PO_4	0.075	0.00025	***
**02**	0	Na_3PO_4	0.075	02	50	Na_3PO_4	0.075	0.00025	***
**02**	1	Na_3PO_4	0.075	02	5	Na_3PO_4	0.075	0.00012	***
**02**	1	Na_3PO_4	0.075	02	10	Na_3PO_4	0.075	0.00026	***
**02**	1	Na_3PO_4	0.075	02	25	Na_3PO_4	0.075	0.00026	***
**02**	1	Na_3PO_4	0.075	02	50	Na_3PO_4	0.075	0.00026	***
**02**	5	Na_3PO_4	0.075	02	10	Na_3PO_4	0.075	0.00086	***
**02**	5	Na_3PO_4	0.075	02	25	Na_3PO_4	0.075	0.00086	***
**02**	5	Na_3PO_4	0.075	02	50	Na_3PO_4	0.075	0.00086	***
**02**	10	Na_3PO_4	0.075	02	25	Na_3PO_4	0.075	Nd	Nd
**02**	10	Na_3PO_4	0.075	02	50	Na_3PO_4	0.075	Nd	Nd
**02**	25	Na_3PO_4	0.075	02	50	Na_3PO_4	0.075	Nd	Nd
**06**	DC	Na_2CO_3	75	06	DC	Na_3PO_4	75	0.84682	Ns
**06**	0	Na_2CO_3	75	06	0	Na_3PO_4	75	0.34206	Ns
**06**	1	Na_2CO_3	75	06	1	Na_3PO_4	75	0.21198	Ns
**06**	5	Na_2CO_3	75	06	5	Na_3PO_4	75	0.10296	Ns

**06**	10	Na_2CO_3	75	06	10	Na_3PO_4	75	0.02327	*
**06**	25	Na_2CO_3	75	06	25	Na_3PO_4	75	0.01373	*
**06**	50	Na_2CO_3	75	06	50	Na_3PO_4	75	0.00413	**
**06**	DC	Na_2CO_3	7.5	06	DC	Na_3PO_4	7.5	0.43976	Ns
**06**	0	Na_2CO_3	7.5	06	0	Na_3PO_4	7.5	0.35449	Ns
**06**	1	Na_2CO_3	7.5	06	1	Na_3PO_4	7.5	0.07791	Ns
**06**	5	Na_2CO_3	7.5	06	5	Na_3PO_4	7.5	0.00001	***
**06**	10	Na_2CO_3	7.5	06	10	Na_3PO_4	7.5	0.00001	***
**06**	25	Na_2CO_3	7.5	06	25	Na_3PO_4	7.5	0.00001	***
**06**	50	Na_2CO_3	7.5	06	50	Na_3PO_4	7.5	0.00419	**
**06**	DC	Na_2CO_3	0.75	06	DC	Na_3PO_4	0.75	0.45761	Ns
**06**	0	Na_2CO_3	0.75	06	0	Na_3PO_4	0.75	0.35417	Ns
**06**	1	Na_2CO_3	0.75	06	1	Na_3PO_4	0.75	0.52560	Ns
**06**	5	Na_2CO_3	0.75	06	5	Na_3PO_4	0.75	0.01458	*
**06**	10	Na_2CO_3	0.75	06	10	Na_3PO_4	0.75	0.13552	Ns
**06**	25	Na_2CO_3	0.75	06	25	Na_3PO_4	0.75	0.31449	Ns
**06**	50	Na_2CO_3	0.75	06	50	Na_3PO_4	0.75	Nd	Nd
**06**	DC	Na_2CO_3	0.075	06	DC	Na_3PO_4	0.075	0.02139	*
**06**	0	Na_2CO_3	0.075	06	0	Na_3PO_4	0.075	0.06364	Ns
**06**	1	Na_2CO_3	0.075	06	1	Na_3PO_4	0.075	0.01837	*
**06**	5	Na_2CO_3	0.075	06	5	Na_3PO_4	0.075	0.02719	*
**06**	10	Na_2CO_3	0.075	06	10	Na_3PO_4	0.075	0.00740	**
**06**	25	Na_2CO_3	0.075	06	25	Na_3PO_4	0.075	0.42265	Ns
**06**	50	Na_2CO_3	0.075	06	50	Na_3PO_4	0.075	0.42265	Ns
**02**	DC	Na_2CO_3	75	02	DC	Na_3PO_4	75	0.01786	*
**02**	0	Na_2CO_3	75	02	0	Na_3PO_4	75	0.24934	Ns
**02**	1	Na_2CO_3	75	02	1	Na_3PO_4	75	0.25026	Ns
**02**	5	Na_2CO_3	75	02	5	Na_3PO_4	75	0.06494	Ns
**02**	10	Na_2CO_3	75	02	10	Na_3PO_4	75	0.05090	Ns
**02**	25	Na_2CO_3	75	02	25	Na_3PO_4	75	0.05478	Ns
**02**	50	Na_2CO_3	75	02	50	Na_3PO_4	75	0.05567	Ns
**02**	DC	Na_2CO_3	7.5	02	DC	Na_3PO_4	7.5	0.00035	***
**02**	0	Na_2CO_3	7.5	02	0	Na_3PO_4	7.5	0.00275	**
**02**	1	Na_2CO_3	7.5	02	1	Na_3PO_4	7.5	0.00006	***
**02**	5	Na_2CO_3	7.5	02	5	Na_3PO_4	7.5	0.01056	*
**02**	10	Na_2CO_3	7.5	02	10	Na_3PO_4	7.5	0.03090	*
**02**	25	Na_2CO_3	7.5	02	25	Na_3PO_4	7.5	0.74593	Ns
**02**	50	Na_2CO_3	7.5	02	50	Na_3PO_4	7.5	0.01365	*
**02**	DC	Na_2CO_3	0.75	02	DC	Na_3PO_4	0.75	0.39303	Ns
**02**	0	Na_2CO_3	0.75	02	0	Na_3PO_4	0.75	0.11565	Ns
**02**	1	Na_2CO_3	0.75	02	1	Na_3PO_4	0.75	0.03428	*
**02**	5	Na_2CO_3	0.75	02	5	Na_3PO_4	0.75	0.01140	*
**02**	10	Na_2CO_3	0.75	02	10	Na_3PO_4	0.75	0.00663	**
**02**	25	Na_2CO_3	0.75	02	25	Na_3PO_4	0.75	0.00221	**
**02**	50	Na_2CO_3	0.75	02	50	Na_3PO_4	0.75	Nd	Nd
**02**	DC	Na_2CO_3	0.075	02	DC	Na_3PO_4	0.075	0.16103	Ns
**02**	0	Na_2CO_3	0.075	02	0	Na_3PO_4	0.075	0.68857	Ns
**02**	1	Na_2CO_3	0.075	02	1	Na_3PO_4	0.075	0.55410	Ns
**02**	5	Na_2CO_3	0.075	02	5	Na_3PO_4	0.075	0.23848	Ns
**02**	10	Na_2CO_3	0.075	02	10	Na_3PO_4	0.075	0.00322	**
**02**	25	Na_2CO_3	0.075	02	25	Na_3PO_4	0.075	0.00326	**

**02**	50	Na_2CO_3	0.075	02	50	Na_3PO_4	0.075	Nd	Nd
**06**	DC	Na_2CO_3	75	02	DC	Na_2CO_3	75	0.00919	**
**06**	0	Na_2CO_3	75	02	0	Na_2CO_3	75	0.07976	Ns
**06**	1	Na_2CO_3	75	02	1	Na_2CO_3	75	0.12660	Ns
**06**	5	Na_2CO_3	75	02	5	Na_2CO_3	75	0.00917	**
**06**	10	Na_2CO_3	75	02	10	Na_2CO_3	75	0.14148	Ns
**06**	25	Na_2CO_3	75	02	25	Na_2CO_3	75	0.08244	Ns
**06**	50	Na_2CO_3	75	02	50	Na_2CO_3	75	0.18930	Ns
**06**	DC	Na_2CO_3	7.5	02	DC	Na_2CO_3	7.5	0.00069	***
**06**	0	Na_2CO_3	7.5	02	0	Na_2CO_3	7.5	0.73140	Ns
**06**	1	Na_2CO_3	7.5	02	1	Na_2CO_3	7.5	0.00234	**
**06**	5	Na_2CO_3	7.5	02	5	Na_2CO_3	7.5	0.00184	**
**06**	10	Na_2CO_3	7.5	02	10	Na_2CO_3	7.5	0.00257	**
**06**	25	Na_2CO_3	7.5	02	25	Na_2CO_3	7.5	0.19376	Ns
**06**	50	Na_2CO_3	7.5	02	50	Na_2CO_3	7.5	0.03155	*
**06**	DC	Na_2CO_3	0.75	02	DC	Na_2CO_3	0.75	0.29148	Ns
**06**	0	Na_2CO_3	0.75	02	0	Na_2CO_3	0.75	0.06353	Ns
**06**	1	Na_2CO_3	0.75	02	1	Na_2CO_3	0.75	0.00190	**
**06**	5	Na_2CO_3	0.75	02	5	Na_2CO_3	0.75	0.01788	*
**06**	10	Na_2CO_3	0.75	02	10	Na_2CO_3	0.75	0.06633	Ns
**06**	25	Na_2CO_3	0.75	02	25	Na_2CO_3	0.75	0.00013	***
**06**	50	Na_2CO_3	0.75	02	50	Na_2CO_3	0.75	Nd	Nd
**06**	DC	Na_2CO_3	0.075	02	DC	Na_2CO_3	0.075	0.07780	Ns
**06**	0	Na_2CO_3	0.075	02	0	Na_2CO_3	0.075	0.14249	Ns
**06**	1	Na_2CO_3	0.075	02	1	Na_2CO_3	0.075	0.00004	***
**06**	5	Na_2CO_3	0.075	02	5	Na_2CO_3	0.075	0.01193	*
**06**	10	Na_2CO_3	0.075	02	10	Na_2CO_3	0.075	0.05814	Ns
**06**	25	Na_2CO_3	0.075	02	25	Na_2CO_3	0.075	0.00326	**
**06**	50	Na_2CO_3	0.075	02	50	Na_2CO_3	0.075	Nd	Nd
**06**	DC	Na_3PO_4	75	02	DC	Na_3PO_4	75	0.67437	Ns
**06**	0	Na_3PO_4	75	02	0	Na_3PO_4	75	0.23400	Ns
**06**	1	Na_3PO_4	75	02	1	Na_3PO_4	75	0.27023	Ns
**06**	5	Na_3PO_4	75	02	5	Na_3PO_4	75	0.28178	Ns
**06**	10	Na_3PO_4	75	02	10	Na_3PO_4	75	0.89060	Ns
**06**	25	Na_3PO_4	75	02	25	Na_3PO_4	75	0.12406	Ns
**06**	50	Na_3PO_4	75	02	50	Na_3PO_4	75	0.34674	Ns
**06**	DC	Na_3PO_4	7.5	02	DC	Na_3PO_4	7.5	0.02280	*
**06**	0	Na_3PO_4	7.5	02	0	Na_3PO_4	7.5	0.00228	**
**06**	1	Na_3PO_4	7.5	02	1	Na_3PO_4	7.5	0.00001	***
**06**	5	Na_3PO_4	7.5	02	5	Na_3PO_4	7.5	0.00011	***
**06**	10	Na_3PO_4	7.5	02	10	Na_3PO_4	7.5	0.00856	**
**06**	25	Na_3PO_4	7.5	02	25	Na_3PO_4	7.5	0.00208	**
**06**	50	Na_3PO_4	7.5	02	50	Na_3PO_4	7.5	0.00538	**
**06**	DC	Na_3PO_4	0.75	02	DC	Na_3PO_4	0.75	0.18597	Ns
**06**	0	Na_3PO_4	0.75	02	0	Na_3PO_4	0.75	0.02329	*
**06**	1	Na_3PO_4	0.75	02	1	Na_3PO_4	0.75	0.00719	**
**06**	5	Na_3PO_4	0.75	02	5	Na_3PO_4	0.75	0.01176	*
**06**	10	Na_3PO_4	0.75	02	10	Na_3PO_4	0.75	0.01098	*
**06**	25	Na_3PO_4	0.75	02	25	Na_3PO_4	0.75	Nd	Nd
**06**	50	Na_3PO_4	0.75	02	50	Na_3PO_4	0.75	Nd	Nd
**06**	DC	Na_3PO_4	0.075	02	DC	Na_3PO_4	0.075	0.04301	*

**06**	0	Na_3PO_4	0.075	02	0	Na_3PO_4	0.075	0.07059	Ns
**06**	1	Na_3PO_4	0.075	02	1	Na_3PO_4	0.075	0.00170	**
**06**	5	Na_3PO_4	0.075	02	5	Na_3PO_4	0.075	0.36641	Ns
**06**	10	Na_3PO_4	0.075	02	10	Na_3PO_4	0.075	0.42265	Ns
**06**	25	Na_3PO_4	0.075	02	25	Na_3PO_4	0.075	0.42265	Ns
**06**	50	Na_3PO_4	0.075	02	50	Na_3PO_4	0.075	0.42265	Ns
