# Supplementary material for: Interplay of phosphate and carbonate ions with flavin photosensitizers in photodynamic inactivation of bacteria
Source: PLoS One. 2021 Jun 11;16(6):e0253212. doi: 10.1371/journal.pone.0253212 (PMC8195418; doi:10.1371/journal.pone.0253212)
Supplement: S5 File — (PDF) [file pone.0253212.s012.pdf]

Statistical analysis of binding assay data

Method

In order to investigate the gathered data statistically, derived from each triplicate means and standard deviation were calculated. Significance between samples was calculated via unpaired, two-tailed t-tests assuming normal distribution. Events were considered statistically significant for $p < 0.05$ and marked in the following table with one asterisk. When p is < 0.01, events were considered highly significant and marked with two asterisks. Extremely significant events with $p < 0.001$ were marked with three asterisks. Non-significant events were marked with "Ns".

Results

Table 1: Results of the performed t-tests for each of the listed conditions concerning the binding assays. The abbreviations in the table indicate the following: Sa: Staphylococcus aurues, Pa: Pseudomonas aeruginosa, 02: FLASH-02a, 06: FLASH-06a. The applied aqueous environment is abbreviated as epimical formula whereas the next column indicates the corresponding concentration in mmol l^{-1}

**Condition 1**				**Vs.**	**Condition 2**				**p**	**significance**
Sa	02	H_2O	-		Sa	02	Na_2CO_3	75	0.69940	Ns
Sa	02	H_2O	-		Sa	02	Na_2CO_3	0.075	0.14525	Ns
Sa	02	H_2O	-		Sa	02	Na_3PO_4	75	0.87630	Ns
Sa	02	H_2O	-		Sa	02	Na_3PO_4	0.075	0.23247	Ns
Sa	02	Na_2CO_3	75		Sa	02	Na_2CO_3	0.075	0.35620	Ns
Sa	02	Na_2CO_3	75		Sa	02	Na_3PO_4	75	0.74105	Ns
Sa	02	Na_2CO_3	75		Sa	02	Na_3PO_4	0.075	0.51980	Ns
Sa	02	Na_2CO_3	0.075		Sa	02	Na_3PO_4	75	0.16179	Ns
Sa	02	Na_2CO_3	0.075		Sa	02	Na_3PO_4	0.075	0.71652	Ns
Sa	02	Na_3PO_4	75		Sa	02	Na_3PO_4	0.075	0.24498	Ns
Sa	06	H_2O	-		Sa	06	Na_2CO_3	75	0.15911	Ns
Sa	06	H_2O	-		Sa	06	Na_2CO_3	0.075	0.01485	*
Sa	06	H_2O	-		Sa	06	Na_3PO_4	75	0.04638	*
Sa	06	H_2O	-		Sa	06	Na_3PO_4	0.075	0.00799	**
Sa	06	Na_2CO_3	75		Sa	06	Na_2CO_3	0.075	0.19346	Ns
Sa	06	Na_2CO_3	75		Sa	06	Na_3PO_4	75	0.58806	Ns
Sa	06	Na_2CO_3	75		Sa	06	Na_3PO_4	0.075	0.00799	**
Sa	06	Na_2CO_3	0.075		Sa	06	Na_3PO_4	75	0.28993	Ns
Sa	06	Na_2CO_3	0.075		Sa	06	Na_3PO_4	0.075	0.46346	Ns
Sa	06	Na_3PO_4	75		Sa	06	Na_3PO_4	0.075	0.11322	Ns
Sa	02	H_2O	-		Sa	06	H_2O	-	0.00230	**
Sa	02	Na_2CO_3	75		Sa	06	Na_2CO_3	75	0.03150	*
Sa	02	Na_2CO_3	0.075		Sa	06	Na_2CO_3	0.075	0.01958	*
Sa	02	Na_3PO_4	75		Sa	06	Na_3PO_4	75	0.02101	*
Sa	02	Na_3PO_4	0.075		Sa	06	Na_3PO_4	0.075	0.04596	*
Pa	02	H_2O	-		Pa	02	Na_2CO_3	75	0.05459	Ns
Pa	02	H_2O	-		Pa	02	Na_2CO_3	0.075	0.00244	**
Pa	02	H_2O	-		Pa	02	Na_3PO_4	75	0.00007	***
Pa	02	H_2O	-		Pa	02	Na_3PO_4	0.075	0.00020	***
Pa	02	Na_2CO_3	75		Pa	02	Na_2CO_3	0.075	0.00178	**
Pa	02	Na_2CO_3	75		Pa	02	Na_3PO_4	75	0.00075	***
Pa	02	Na_2CO_3	75		Pa	02	Na_3PO_4	0.075	0.00349	**
Pa	02	Na_2CO_3	0.075		Pa	02	Na_3PO_4	75	0.00118	**
Pa	02	Na_2CO_3	0.075		Pa	02	Na_3PO_4	0.075	0.68979	Ns

Pa	02	Na_3PO_4	75	Pa	02	Na_3PO_4	0.075	0.00050	***
Pa	06	H_2O	-	Pa	06	Na_2CO_3	75	0.47569	Ns
Pa	06	H_2O	-	Pa	06	Na_2CO_3	0.075	0.05298	Ns
Pa	06	H_2O	-	Pa	06	Na_3PO_4	75	0.96189	Ns
Pa	06	H_2O	-	Pa	06	Na_3PO_4	0.075	0.02436	*
Pa	06	Na_2CO_3	75	Pa	06	Na_2CO_3	0.075	0.03974	*
Pa	06	Na_2CO_3	75	Pa	06	Na_3PO_4	75	0.62367	Ns
Pa	06	Na_2CO_3	75	Pa	06	Na_3PO_4	0.075	0.02436	*
Pa	06	Na_2CO_3	0.075	Pa	06	Na_3PO_4	75	0.16308	Ns
Pa	06	Na_2CO_3	0.075	Pa	06	Na_3PO_4	0.075	0.07512	Ns
Pa	06	Na_3PO_4	75	Pa	06	Na_3PO_4	0.075	0.07879	Ns
Pa	02	H_2O	-	Pa	06	H_2O	-	0.03209	*
Pa	02	Na_2CO_3	75	Pa	06	Na_2CO_3	75	0.77612	Ns
Pa	02	Na_2CO_3	0.075	Pa	06	Na_2CO_3	0.075	0.00076	***
Pa	02	Na_3PO_4	75	Pa	06	Na_3PO_4	75	0.00578	**
Pa	02	Na_3PO_4	0.075	Pa	06	Na_3PO_4	0.075	0.00023	***
Sa	02	H_2O	-	Pa	02	H_2O	-	0.54208	Ns
Sa	02	Na_2CO_3	75	Pa	02	Na_2CO_3	75	0.06029	Ns
Sa	02	Na_2CO_3	0.075	Pa	02	Na_2CO_3	0.075	0.08897	Ns
Sa	02	Na_3PO_4	75	Pa	02	Na_3PO_4	75	0.00005	***
Sa	02	Na_3PO_4	0.075	Pa	02	Na_3PO_4	0.075	0.05598	Ns
Sa	06	H_2O	-	Pa	06	H_2O	-	0.09115	Ns
Sa	06	Na_2CO_3	75	Pa	06	Na_2CO_3	75	0.45420	Ns
Sa	06	Na_2CO_3	0.075	Pa	06	Na_2CO_3	0.075	0.30577	Ns
Sa	06	Na_3PO_4	75	Pa	06	Na_3PO_4	75	0.72675	Ns
Sa	06	Na_3PO_4	0.075	Pa	06	Na_3PO_4	0.075	0.09986	Ns
